# The novel synthesis of a condensed triazine *via* the heterocyclization of an azo derivative and its characterization, radiolabeling and bio-evaluation

**DOI:** 10.1039/d5ra05853h

**Published:** 2025-10-21

**Authors:** Wael Shehta, Doaa A. Elsayed, F. Marzook, Mohammed G. Assy, Mahmoud M. Sultan, S. El-Kalyoubi, M. Korany, Mohamed E. Abdu, Mohamed Abdel-Haleem, Mohamed Taha Yassin

**Affiliations:** a Department of Chemistry, Faculty of Science, Zagazig University Zagazig 44519 Egypt doaaatef641995@gmail.com doaaatef@zu.edu.eg; b Labelled Compounds Department, Hot Labs Center, Egyptian Atomic Energy Authority P.O. Box 13759 Cairo Egypt; c Pharmaceutical Organic Chemistry, Faculty of Pharmacy, PortSaid University Egypt; d Department of Interdisciplinary Engineering Sciences, Chemistry and Materials Science, Interdisciplinary Graduate School of Engineering Sciences, Kyushu University 6-1 Kasuga Park Fukuoka 816-8580 Japan; e Department of Botany and Microbiology, Faculty of Science, Zagazig University 44519 Zagazig Egypt; f Department of Botany and Microbiology, College of Science, King Saud University P.O. Box 2455 Riyadh 11451 Saudi Arabia

## Abstract

In heterocyclic chemistry, many studies have adopted the synthesis of novel benzotriazinone derivatives, as they have shown significant potential in biomedical research, particularly as enzyme inhibitors. This makes benzotriazinone a valuable scaffold for the development of new pharmaceuticals. Herein, a novel series of benzotriazinone derivatives was successfully synthesized, followed by docking studies. Ethyl 2-carboxyphenylazocyanoacetate was synthesized through the reaction of one mole of diazonium salt with a percentage of a mole of ethyl cyanoacetate in an alkaline medium. One of the derivatives, Triazine 12, exhibited promising *in silico* antitumor activity, according to molecular docking studies, which was then confirmed *in vitro* by determining its cytotoxicity against the human tumor cell lines HepG-2 and MCF-7, with IC_50_ values of 78.53 ± 3.49 µg ml^−1^ and 48.31 ± 2.37 µg ml^−1^, respectively, in addition to low cytotoxicity against normal lung fibroblast cells (MRC-5), with a moderate antioxidant capacity shown in the DPPH radical scavenging assay. Furthermore, its suitability for radiolabeling as a tracer for *in vivo* studies was checked. Triazine 12 was directly radiolabeled with technetium-99m (^99m^Tc), yielding a radiochemical purity of 95.4% ± 0.46%. A biodistribution study in tumor-bearing mice of [^99m^Tc]-labeled Triazine 12 exhibited significant tumor targeting properties with positive T/NT ratios, reaching a peak of 4.65 (more than many tumoral radiopharmaceuticals) 1-hour post-injection, which highlights its potential as a novel radiotracer for tumor imaging.

## Abstract 2

New benzotriazinone derivatives were synthesized. Triazine 12 showed strong antitumor activity, low toxicity, and efficient ^99m^Tc labeling with high tumor uptake, suggesting potential as a tumor imaging agent.

## Graphical abstract

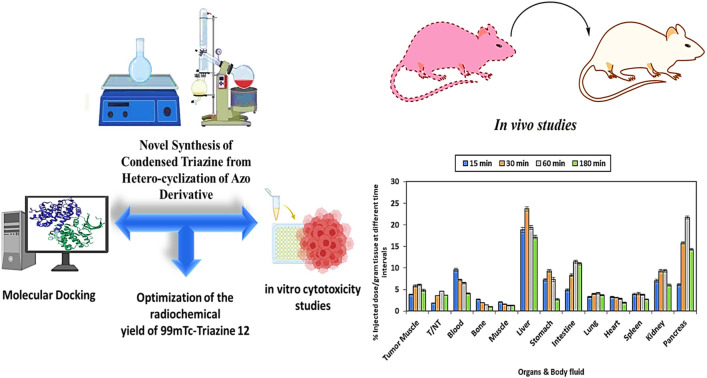

## 1. Introduction

The current research in heterocyclic chemistry is pertinent to developing new synthetic methods for ring synthesis. In recent years, there has been growing interest in the field of chemistry concerning *vic*-benzotriazinone derivatives, due to their wide range of biological activities, including anticancer,^1^ antimicrobial,^2^ antiviral,^3^ analgesic,^4^ anti-inflammatory,^5,6^ antimicrobial,^7,8^ antihistaminic,^9^ antiangiogenic,^10^ and antifungal.^11^ Some analogues have demonstrated effective pharmacologic activity and are considered promising molecules for developing futuristic drugs such as *v*-triazines or *vic*-triazines. They are acknowledged as the least investigated examples to date, although the ring system is characterized by its stability. There are no reports associated with biological data concerning monocyclic 1,2,3-triazines nor the benzo- and hetero-fused 1,2,3-triazine derivatives.

Molecular docking studies are conducted to fully understand the atomic-level interactions between the protein and the drug/ligand, according to Hamed, *et al.*^12^ The *in silico* information analysis can help explain the fundamental biological processes and characterize how these drugs behave at target binding sites. Using Molecular Operating Environment (MOE, ver. 2022), all of the conformational syntheses will be docked with the enzymes (5V67, 1RR8, 3IG7, 4ASD, 3FC4, 1QZR, and 1YET).^13–15^ These substances may also facilitate exploring and demonstrating the use of newly synthesized therapeutic and preventative drugs for the treatment of cancer-related diseases.

This study aims to synthesize and estimate a series of heterocyclic compounds that may have a potential impact on cancer research development, particularly in the synthesis of novel targeted cancer therapy and imaging agents. This study relies on deep *in silico* screening, followed by selecting the most potent benzotriazinone derivative according to the *in silico* investigation, then radiolabeling with technetium-99m (^99m^Tc). In addition to evaluating its tumoral bioselectivity in mice, we assessed the *in vitro* antitumoral bioactivity of the most promising *in silico* potent benzotriazinone derivatives.^16,17^

[^99m^Tc]-Radiolabeling of the selected benzotriazinone derivative may represent a possible novel probe in the development of next-generation radiopharmaceuticals, according to the expected results of radiolabeling purity, stability, and biodistribution. Accordingly, the current study aims to screen the traceability and specificity of the *in silico* selected compound, and assess its ability for real-time monitoring and precise localization of tumor sites *in vivo*, clarifying its potential impact in the future clinical setting. Furthermore, studying the molecular interactions underlying tumor targeting is important for understanding the therapeutic impacts of benzotriazinones in cancer management.^18–22^ The integration of synthetic chemistry with radiochemistry and molecular imaging studies creates potential for the implementation of novel antitumor radiopharmaceutical probes with enhanced tumor targeting capabilities and improved therapeutic results. These results are expected to be a step forward in both synthetic methodology and molecular benzotriazinone's contribution to nuclear medicine, following the new strategies for cancer treatment and diagnosis.^23–26^

Benzotriazines are heterocyclic compounds comprising benzene rings fused to triazine rings. These groups of compounds have gathered interest in anticancer research due to their capability to inhibit cancer cell growth, induce apoptosis, and target specific molecular pathways involved in tumor development.^27–30^ Temozolomide (TMZ) is a well-known, very active triazine-based drug possessing strong antitumor properties used in the treatment of glioblastoma multiforme (a highly aggressive brain tumor) and other cancers.^31–33^

**Fig. 1:**
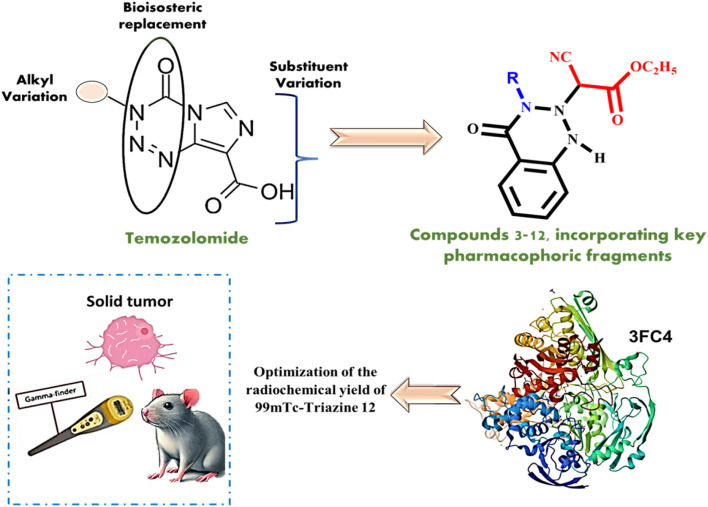
The design of novel condensed azines and triazines, along with the characterization, radiolabeling and further bioevaluation of Triazine 12.

A number of our synthesized compounds based on the temozolomide structural framework were assessed for their anticancer properties. Molecular docking was used to study the anti-tumor activity of the produced compounds, and *in silico* docking studies indicated that compound 12 has promising anti-tumor activity, which was subsequently confirmed through practical application against HepG-2 and MCF-7 human cancer cell lines. To complement the tumor-targeting and radiolabeling studies, we further assessed the safety and ancillary bioactivity of Triazine 12, including its cytotoxicity against normal human fibroblast cells and its antioxidant potential, providing a broader view of its pharmacological profile. Furthermore, the potential of the promising candidate 12 for radiolabeling was evaluated as a possible tracer and investigated in *in vivo* studies. The tested compound is expected to be a successful candidate for radiolabeling with technetium-99m (^99m^Tc), which permits its further consideration in preclinical and clinical settings as a precise tool for cancer imaging and therapy, Fig. 1.

## 2. Results and discussion

### 2.1. Chemistry

**Fig. 2:**
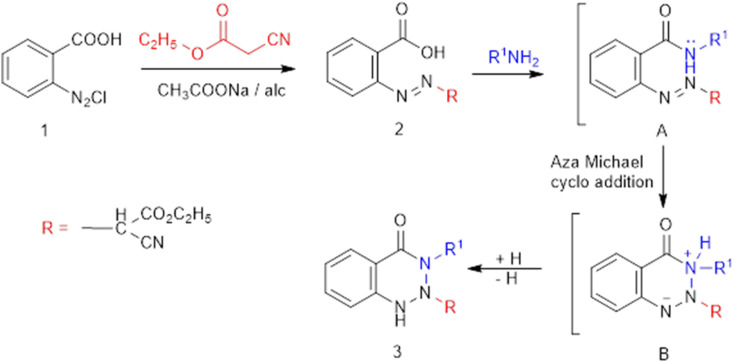
The new route to a novel benzotriazinone class.

The innovatively designed *vic*-benzotriazinone derivatives were prepared from ethyl 2-carboxyphenylazocyanoacetate (2) as precursor through a newly developed route. The chemical structure of the azo structure of 2 was confirmed by the existence of a singlet signal at 2.46 ppm, in addition to stretching frequencies of CN and C=O at 2210 and (1736, 1666) cm^−1^, respectively. The synthetic approach to *v*-benzotriazinone includes two main steps: The initial step involves the condensation of a nitrogen amino nucleophilic reagent with the activated electrophilic carbon of the carboxylic group (–M of the phenylazo moiety). The second involves intramolecular aza-Michael cycloaddition of the imino functionality to the polarized N=N–Ar, as shown in general Fig. 2.

Refluxing 2 with bidentate nucleophiles, namely, hydrazine, phenylhydrazine, and/or guanidine, affords 2-hydroxy-10-oxo-5,10-dihydro-3*H*-benzo[*d*][1,2,3]triazolo[2,1-*a*][1,2,3]triazine-3-carbonitrile (4), ethyl 2-cyano-2-(4-oxo-3-(phenylamino)-3,4-dihydrobenzo[*d*][1,2,3]triazin-2(1*H*)-yl) acetate (5), and ethyl 2-cyano-2-(3-cyano-4-oxo-3,4-dihydrobenzo[*d*][1,2,3]triazin-2(1*H*)-yl)acetate (6), respectively (Fig. 2). Compound 4 was thought to be formed through the initial condensation of 2 with hydrazine to afford non-isolable intermediate B, which undergoes aza-Michael cycloaddition to give Michael adduct C, followed by intramolecular cyclocondensation with loss of ethanol and the subsequent tautomerism to the enolic form 4.

The IR spectrum of benzotriazolotriazine 4 demonstrates characteristic absorption bands for OH, NH, and CN at 3444, 3239, and 2225 cm^−1^, respectively. The chemical structure of the poly-heterocyclic derivative 4 was further confirmed by ^1^H NMR spectroscopy, as it showed downfield characteristics of OH and triazine NH as a singlet at 14.24 and 10.47 ppm, as well as the disappearance of the ester protons. Similarly, benzotriazinone 5 was obtained, which failed to undergo further heterocyclocondensation as a result of involving the acidic amino NH in the formation of an intramolecular H-bond.

**Fig. 3:**
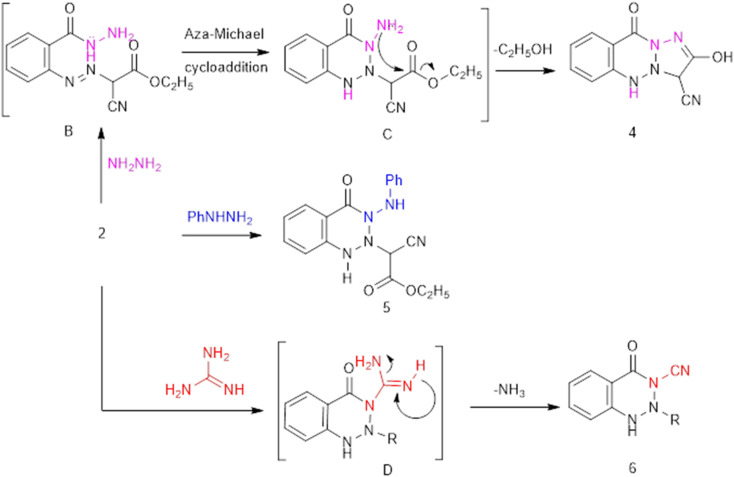
The synthesis of condensed triazine.

The IR spectrum of 5 showed absorption bands at 3247 (broad), 2233, 1704, 1602 cm^−1^, corresponding to NH, CN, and CO, respectively. In addition, the ^1^H NMR spectrum showed signals for heterocyclic NH and imino protons at 13.07 and 14.53 ppm, respectively, in addition to signals for the ethyl protons. On the other hand, the *N*-cyano-*v*-triazinone 6 was obtained *via* formation of non-isolable triazine D, followed by loss of ammonia. The structure of 6 was ascertained by IR, which exhibited double cyano-absorption bands together with the expected frequencies for NH and CO at 3448, 1704, and 1592 cm^−1^, and by the ^1^H NMR, which showed a singlet signal at 15.4 ppm assigned to NH triazinone (Fig. 3).

**Fig. 4:**
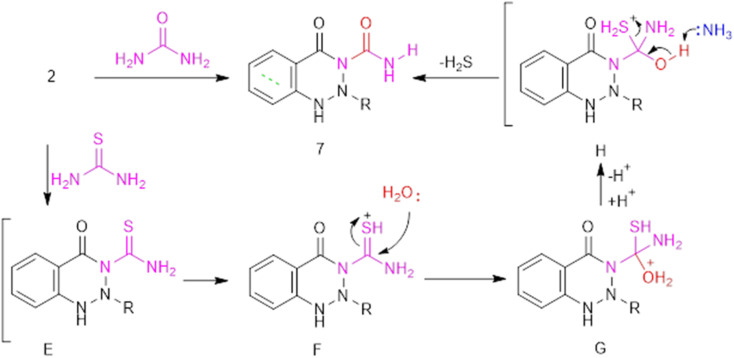
The formation of triazinone *N*-carboxyamide 7.

The benzotriazine *N*-carboxamide derivative 7 was obtained *via* the reaction of dipolarized compound 2 with urea and/or thiourea (Fig. 4). The IR spectrum of 7 exhibits stretching frequencies at 3436, 2229, and (1690, 1647) cm^−1^, assigned to NH, CN, and CO groups, respectively. The chemical structure of 7 was further confirmed by ^1^H NMR spectroscopy, which displays a broad singlet signal at 6.78 ppm assigned to NH_2_ amide (which forms an H-bond with CO) and 7.72 ppm assigned to NH triazine. Furthermore, the ^13^C NMR spectrum showed signals at 169.52, 168.80, 162.82, and 148.60 ppm, corresponding to carbonyl and cyanocarbons, respectively. On the other hand, the formation of 7 through the reaction with thiourea was assumed to go through *in situ* generation of non-isolable intermediates E-H, which involves the addition of water and subsequent loss of H_2_S gas.

**Fig. 5:**
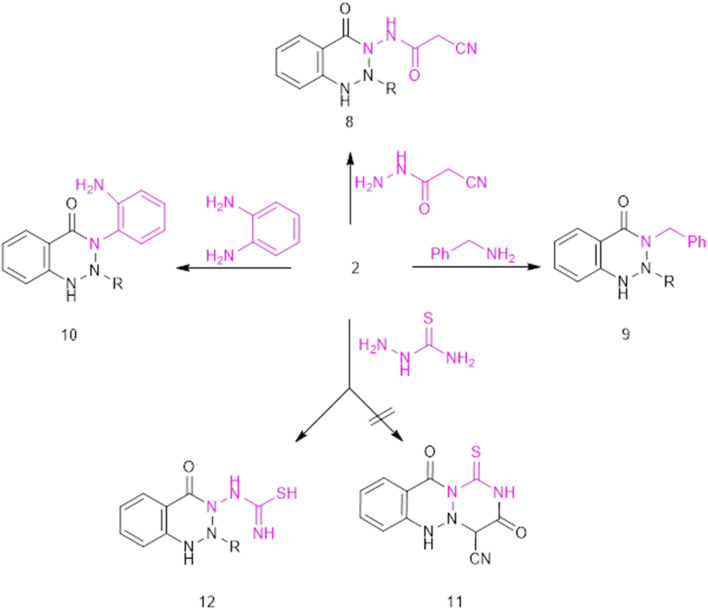
The behavior of the 2-carboxyphenylazocyanoacetate derivative 2 towards nitrogen nucleophiles with different moieties.

Under identical thermal conditions, the reaction of 2 with cyanoacetohydrazide afforded ethyl 2-cyano-2-(3-(2-cyanoacetamido)-4-oxo-3,4-dihydrobenzo[*d*][1,2,3]triazin-2(1*H*)-yl)acetate 8 (Fig. 5). The IR spectrum showed absorption peaks at 3216, 3166, 2210, and (1739, 1697) cm^−1^, assigned to two NH, two CN, and C=O, respectively. The ^1^H NMR exhibits key signals at 5.84 and 7.96 ppm, assigned to *v*-triazinone and amide NH, respectively. In the ^13^C-NMR spectrum, carbonyl and cyano-carbon signals were shown at 169.30, 163.74, and 150.73 ppm, respectively, as well as the sp^3^ carbons that showed their resonation upfield. Additionally, treating 2 with benzylamine resulted in the production of *N*-benzylbenzotriazinone derivative 9. The IR spectrum of 9 included NH, CN, and CO at 3432, 2210, (1739, 1693) cm^−1^, respectively, and the ^13^C-NMR of compound 9 shows signals at 169.27, 163.70, and 150.66 for CO and nitrile carbon, in addition to the sp^3^ carbon at 31.03 and 19.24 ppm.

As shown in Fig. 5, reaction of compound 2 with phenylenediamine resulted in a condensation process followed by Michael addition for the sake of activating the azo function, and subsequent triazine cyclization afforded ethyl 2-(3-(2-aminophenyl)-4-oxo-3,4-dihydrobenzo[*d*][1,2,3]triazin-2(1*H*)-yl)-2-cyanoacetate (10). The IR spectrum displayed stretching frequencies at 3417–3359, 2225, and (1735, 1689) cm^−1^, assigned to NH, CN, and CO groups, respectively. The ^1^H NMR spectrum exhibited two singlet signals at 5.1 and 14.7 ppm for NH_2_ and heterocyclic NH, respectively. The ^13^C NMR spectrum showed some signals at *δ* 169.44 and 162.76 ppm; in contrast, an observation of cyano-carbon was introduced at *δ* 148.70 ppm. Furthermore, polycyclic triazine derivative 12 was obtained as a result of the reaction of 2 with thiosemicarbazide, which failed to undergo further cyclization to afford polycyclic derivative 11. Target 12 showed CN and CO peaks at 2210 and (1697) cm^−1^, respectively. The chemical structure of 12 was confirmed by ^1^H NMR spectroscopy, which showed four singlet signals at 1.91, 6.79, 12.28, and 12.75 ppm, assigned to SH, cyclic NH, NH, and imine NH, respectively, in addition to signals for ethoxy protons.

**Fig. 6:**
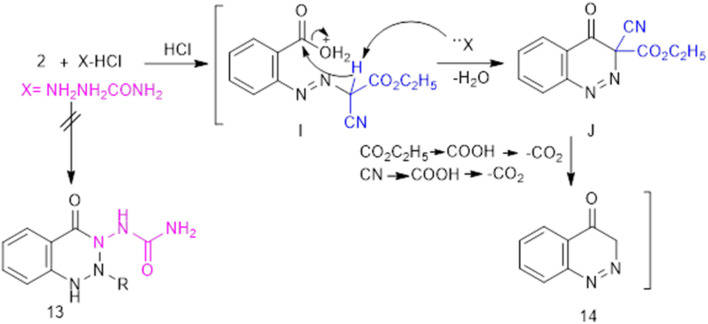
The novel synthesis of cinnolinone 14.

Lastly, cinnoline derivative 14 was produced as an unexpected product by the reaction of 2-carboxyphenylazocyanoacetate 2 with semicarbazide hydrochloride instead of *vic*-triazinone with *N*-urea derivative 13 (Fig. 6). As a new method for cinnolinone 14, it was assumed to proceed *via* the acid-base catalysis of semicarbazide hydrochloride, through the generation of intermediate I, which undergoes intramolecular nucleophilic cyclization to afford J, followed by hydrolysis of the ester and cyano groups and subsequent decarboxylation. Spectral data of 14 led to the absorptive beak of CO expanding at 1681 cm^−1^ and a signal at *δ* = 5.88–7.57.

### 2.2. Molecular docking

The study of molecular docking was carried out using MOE (ver. 2022) to verify the interaction between synthetic chemicals and targets related to anti-cancer disease. All 10 of the targets linked to anti-cancer disease were individually docked with each of the drugs. With regard to all 10 anti-cancer related targets, these substances exhibited extremely high binding affinities. Just two out of all the compounds interacted with the maximum number of targets. Compounds 8 and 12 interacted with six targets (5V67, 3IG7, 4ASD, 3FC4, 1QZR, and 1YET), Table 1. The range of binding affinities pertinent to all compounds linked to anti-cancer disease, particularly with aldehyde oxidoreductase (PDB code: 3FC4), lies between −5.7383 and −9.5621 kcal mol^−1^. Furthermore, the residues, which surrounded the ligands (in small RMSD) in all docking results, contribute to prevailing short-range polar interactions stabilizing the complex formation (in the aid of charge delocalization on ligands). It is not feasible to figure out all binding conformations; however, this study has demonstrated four conformations with the strongest binding affinity with 3FC4, which were compared to the co-crystallized ligand (molybdopterin-cytosine dinucleotide-*s*,*s*)-dioxo-aqua-molybdenum(v) through docking into the effective site, while implementing similar parameters to validate the present docking study of the active site.

**Table 1:** The binding scores and RMSD values of the promising anti-cancer compounds

PDB ID														
	5V67		1RR8		3IG7		4ASD		3FC4		1QZR		1YET	
	Score (kcal mol^−1^)	RMSD	Score (kcal mol^−1^)	RMSD	Score (kcal mol^−1^)	RMSD	Score (kcal mol^−1^)	RMSD	Score (kcal mol^−1^)	RMSD	Score (kcal mol^−1^)	RMSD	Score (kcal mol^−1^)	RMSD
12	−7.211	0.8935	−7.8361	17 067	−6.9946	0.8957	−6.74197	1.689	**−8.7283**	1.3618	**−6.7332**	1.6924	−6.4635	1.5358
8	−6.9284	1.5901	−5.9861	1.6364	−7.18976	1.1265	−7.18251	1.6499	**−9.28691**	0.9073	−7.3748	1.9201	−7.2933	1.0306
2	−6.6040	1.5735	−6.3706	1.1319	−6.3612	2.0843	−6.2603	1.4318	−7.9195	1.3213	−6.5214	1.3660	−6.2727	0.8718
3	−7.0294	1.9367	−6.1168	0.8110	−7.0498	1.9233	−7.4056	1.2388	−8.9143	1.5986	−7.6113	1.4739	−6.7470	1.3382
4	−6.2604	1.3472	**−6.9959**	1.2576	−5.4549	1.2561	−5.4190	0.8969	−6.7456	1.6310	−5.4448	1.5262	−5.4070	1.7106
5	**−7.2369**	1.3346	−6.4530	2.1238	−7.0273	1.6022	−6.9381	2.0629	−9.1848	1.9435	−7.1249	1.8278	−6.5909	1.5284
6	−6.7340	1.2176	−5.6017	1.2302	−6.7219	1.9762	−6.4482	1.4643	−8.3154	1.2948	−6.7586	1.8129	−6.3797	1.6186
7	−6.6745	1.3605	−5.7962	1.8047	−6.4043	1.3313	−6.7470	1.6825	−8.1039	0.9404	−6.6516	1.4207	−6.3700	0.9138
9	−6.5148	1.5319	−6.1144	1.7216	−7.0671	1.0230	−7.1146	1.0774	−9.1465	1.6223	−7.3653	1.9089	−6.8975	1.1542
10	−6.9402	1.2772	−5.9577	1.7040	−7.1801	1.4220	−6.6995	1.77470	−8.6831	1.6864	−7.0375	1.8876	−6.4963	1.9644

**Fig. 7:**
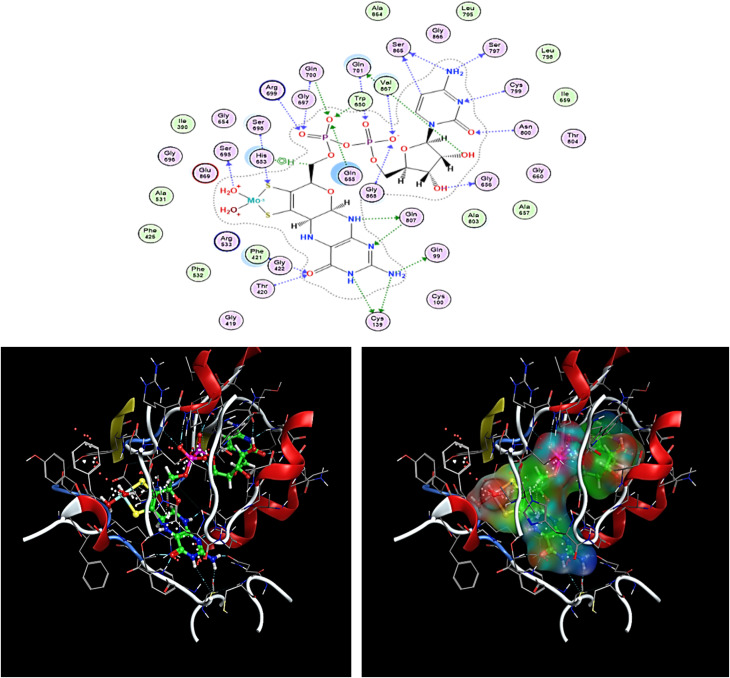
Two- and three-dimensional illustrations of volasertib (green) at the aldehyde oxidoreductase binding site (PDB ID: 3FC4).

The most optimal-docked conformation site identifies a RMSD of 1.1497 Å. The energetic score is −16.6162 kcal mol^−1^, as provided by the docking study with MOE software. Moreover, it formed hydrogen bonds with SER 865 (A), SER 797 (A), SER 865 (A), GLN 701 (A), GLY 656 (A), GLN 807 (A), GLN 99 (A), CYS 139 (A), CYS 139 (A), SER 695 (A), CYS 799 (A), ASN 800 (A), GLN 701 (A), VAL 867 (A), GLY 868 (A), ARG 699 (A), GLN 700 (A), TRP 650 (A), GLN 655 (A), GLN 700 (A), SER 698, GLN 807 (A), THR 420 (A), and PHE 421 (A), although it interacted with pi–H of the 5-ring HIS 653 (A), shown in Fig. 7.

**Fig. 8:**
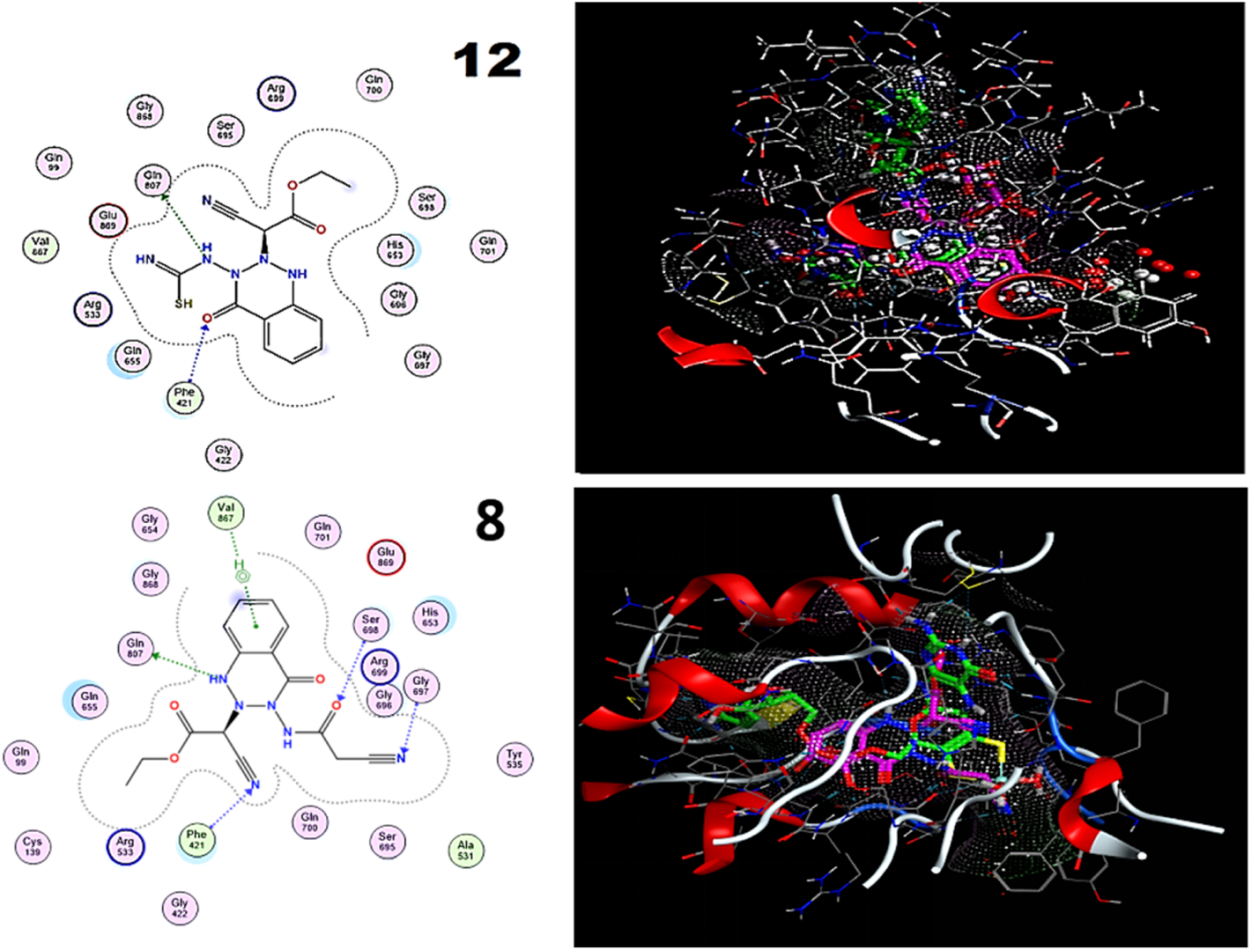
Compounds 12 and 8 (purple) and volasertib (green), showing two-dimensional representations and alignment with the aldehyde oxidoreductase binding site (PDB ID: 3FC4).

Comparing compounds 12 and 8 with reference to the ligand, Table 2 displays the scores of docking studies and the amino acids involved in the interactions with the aldehyde oxidoreductase binding site (PDB ID: 3FC4). Alignment of compounds 12 and 8 with a reference ligand at the aldehyde oxidoreductase binding site (PDB ID: 3FC4) is depicted in Fig. 8. Triazine 12 comprised an interaction of a bifurcated H-bond with PHE 421 (A) and GLN 807. In sharp contrast, compound 8 comprised an interaction of an H-bond with GLN 807(A), GLY 697 (A), SER 698 (A), and PHE 421 (A), also forming a Pi–H bond with VAL 867 (A).

**Table 2:** The binding scores, RMSD values, distances, and receptor interactions of the expected compounds with a comparison to the reference ligand as an anti-cancer marker

Comp.	Score (kcal mol^−1^)	RMSD	Ligand	Receptor	Interactions	Distance (Å)	*E* (kcal mol^−1^)
Ref.	−16.6162	1.1497	C5 5	O SER 865 (A)	H–Donor	3.02	−0.5
			N4 10	O SER 797 (A)	H–Donor	2.78	−3.3
			N4 10	O SER 865 (A)	H–Donor	2.90	−2.0
			O2′ 17	OE1 GLN 701 (A)	H–Donor	2.72	−2.0
			O3′ 24	O GLY 656 (A)	H–Donor	2.82	−0.5
			N8′ 55	OE1 GLN 807 (A)	H–Donor	2.72	−3.4
			N2′ 60	OE1 GLN 99 (A)	H–Donor	2.87	−3.8
			N2′ 60	SG CYS 139 (A)	H–Donor	3.16	−1.7
			N3′ 63	SG CYS 139 (A)	H–Donor	3.94	−0.6
			OM1 71	O SER 695 (A)	H–Donor	3.17	−0.9
			N3 3	N CYS 799 (A)	H–Acceptor	3.02	−2.9
			O2 9	N ASN 800 (A)	H–Acceptor	2.80	−5.3
			O1A 31	N GLN 701 (A)	H–Acceptor	2.82	−5.6
			O2A 32	N VAL 867 (A)	H–Acceptor	2.77	−6.1
			O2A 32	N GLY 868 (A)	H–Acceptor	2.74	−5.9
			O1B 35	N ARG 699 (A)	H–Acceptor	3.37	−0.6
			O1B 35	N GLN 700 (A)	H–Acceptor	3.18	−3.6
			O2B 36	NE1 TRP 650 (A)	H–Acceptor	2.90	−4.9
			O2B 36	NE2 GLN 655 (A)	H–Acceptor	2.85	−5.1
			O2B 36	CB GLN 700 (A)	H–Acceptor	3.423.81	−0.5
			S8′ 44	N SER 698 (A)	H–Acceptor		−3.6
			N1′ 58	NE2 GLN 807 (A)	H–Acceptor	3.04	−4.3
			O4′ 66	N THR 420 (A)	H–Acceptor	2.77	−6.0
			O4′ 66	N PHE 421 (A)	H–Acceptor	2.72	−5.1
			C10 38	5-Ring HIS 653 (A)	H–Pi	4.43	−0.5
12	−8.7283	1.3618	N 4	OE1 GLN 807 (A)	H–Donor	3.04	−1.6
			O 1	CA PHE 421 (A)	H–Acceptor	3.40	−0.6
8	−9.28691	0.9073	N 28	OE1 GLN 807 (A)	H–Donor	3.31	−1.8
			N 11	N GLY 697 (A)	H–Acceptor	3.22	−2.7
			O12	N SER 698 (A)	H–Acceptor	3.08	−1.7
			N 27	CA PHE 421 (A)	H–Acceptor	3.35	−0.6
			6-Ring	N VAL 867 (A)	Pi–H	4.89	−0.7

### 2.3. Assessment of the antioxidant activity of Triazine 12

The percentage radical scavenging activity (RSC%) of Triazine 12 was determined to be 40.6 ± 1.5% compared to the standard ascorbic acid, revealing a quite moderate antioxidant potency for Triazine 12 according to the DPPH assay.

### 2.4. Evaluation of *in vitro* cytotoxicity

**Fig. 9:**
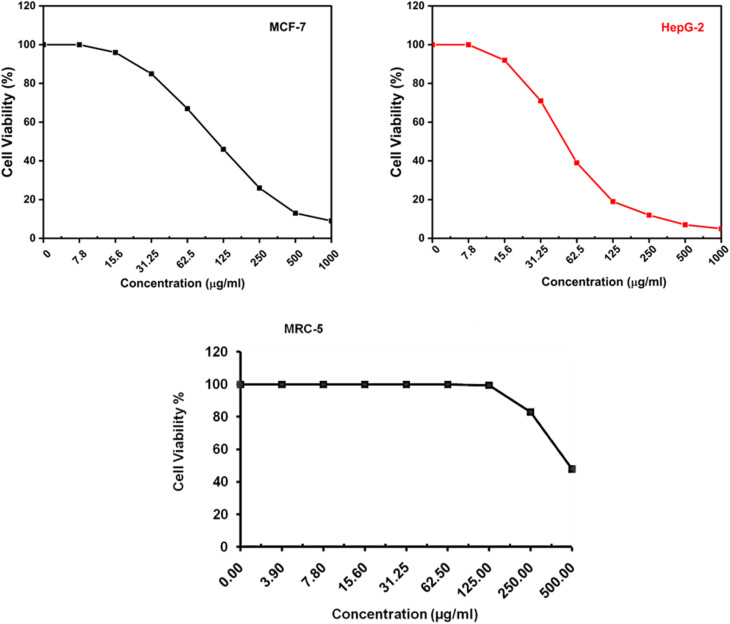
The cytotoxic effect of Triazine 12 on MCF-7 and HepG-2 cell lines based on viability assays, showing an IC_50_ of 78.03 ± 3.49 µg ml^−1^ and an IC_50_ of 48.31 ± 2.37 µg ml^−1^, respectively, in comparison to the low cytotoxicity toward normal human lung fibroblast cells (MRC-5).

The cytotoxic activity of Triazine 12 was studied against both breast carcinoma (MCF-7) and hepatocellular carcinoma (HepG-2) cell lines using a viability assay (MTT) to investigate which of them would be affected more. Fig. 9 showed inhibitory concentrations (IC_50_) of Triazine 12 against MCF-7 and HepG-2, which were 78.53 ± 3.49 and 48.31 ± 2.37 µg ml^−1^, respectively. While the IC_50_ value of 485.16 ± 13.71 µg ml^−1^ for Triazine 12 against normal human lung fibroblast cells was low, the maximum inhibitory suppression was observed up to 52.09% for Triazine 12 at a concentration of 500 µg ml^−1^, as shown in Fig. 9.

### 2.5. Investigation of the cytotoxic mechanism of action of Triazine 12

According to the cytotoxicity results, Triazine 12 demonstrated more cytotoxic activity against HepG-2, so some biomarkers were analyzed to investigate the cytotoxic mechanism of action of Triazine 12 against HepG-2. Tables 3–6 shows the results of the apoptotic markers caspase 3 and Bax in addition to the anti-apoptotic marker Bcl-2, analyzed in triplicate, in Triazine 12-treated HepG-2 cell lines using ELISA. Notably, a great increase in caspase 3 and Bax levels, but a decrease in Bcl-2 levels, was observed in HepG2 cells after treatment with Triazine 12.

**Table 3:** Data analysis of caspase 3 levels (*n* = 3) in HepG-2 cells treated with Triazine 12 at the IC_50_ concentration using ELISA

Sample code	Tested conc. (µg ml^−1^)	Caspase 3 level (ng ml^−1^)			Mean caspase level (ng ml^−1^)	Standard deviation (±)
		1st reading	2nd reading	3rd reading		
Triazine 12 (treated cells)	48.31	78.91	84.27	82.75	81.98	2.76
HepG2 cells (control)	0	44.36	43.59	41.82	43.26	1.30

**Table 4:** Data analysis of Bax levels (*n* = 3) of HepG-2 cells treated with Triazine 12 at the IC_50_ concentration using ELISA

Sample code	Tested conc. (µg ml^−1^)	Bax level (ng ml^−1^)			Mean Bax level (ng ml^−1^)	Standard deviation (±)
		1st reading	2nd reading	3rd reading		
Triazine 12 (treated cells)	48.31	5.77	5.32	5.46	5.52	0.23
HepG2 cells (control)	0	2.31	2.24	2.39	2.31	0.08

**Table 5:** Data analysis of Bcl-2 levels (*n* = 3) of HepG-2 cells treated with Triazine 12 at the IC_50_ concentration using ELISA

Sample code	Tested conc. (µg ml^−1^)	Bcl-2 level (ng ml^−1^)			Mean BCl2 level (ng ml^−1^)	Standard deviation (±)
		1st reading	2nd reading	3rd reading		
Triazine 12 (treated cells)	48.31	3.15	3.47	3.08	3.23	0.21
HepG2 cells (control)	0	6.04	5.91	5.86	5.94	0.09

**Table 6:** Bax/Bcl-2 ratio

Sample code	Tested conc. (µg ml^−1^)	Mean Bax level (ng ml^−1^)	Mean Bcl-2 level (ng ml^−1^)	Bax/Bcl-2
Triazine 12 (treated cells)	48.31	5.52	3.23	1.71
HepG2 cells (control)	0	2.31	5.94	0.39

Further analysis of the mechanism of action against HepG-2 cells at the IC_50_ value of 48.31 ± 2.37 µg ml^−1^ exhibited that this cytotoxic effect is mediated through the induction of apoptosis. Clearly, data from the above tables revealed that compound 12 may significantly contribute to an accumulation of reactive oxygen species (ROS) and subsequent activation of the pro-apoptotic protein Bax. The upregulation of Bax was accompanied by down-regulation of anti-apoptotic protein Bcl-2, which is known to inhibit the activity of Bax. The resulting imbalance between Bax and Bcl-2 (Table 6) entailed cytochrome c release with respect to the mitochondria, stimulating caspase 3 activation and ultimately resulting in apoptosis.

### 2.6. Optimization of the radiochemical yield of [^99m^Tc]-Triazine 12

**Fig. 10:**
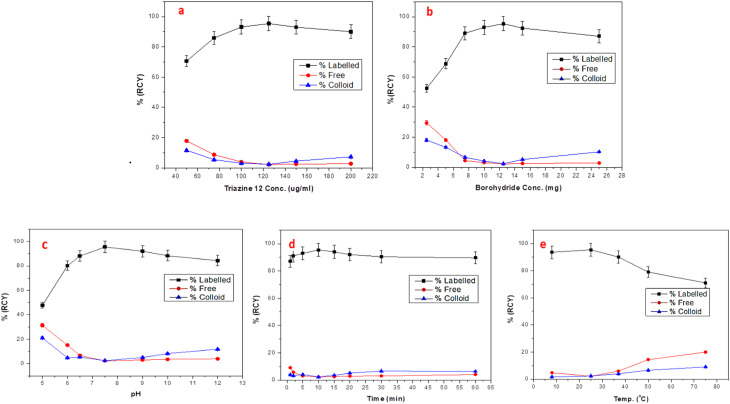
Different effects of (a) substrate concentration, (b) NaBH_4_ concentration, (c) pH, (d) time, and (e) temperature on RCY% of Triazine 12.

The radiolabeling reaction was optimized for concentrations of Triazine 12 and borohydride (the reducing agent), pH, reaction time, and temperature (Fig. 10). The maximum RCY% obtained as determined by paper chromatography technique was 95.4 ± 1.5 by using 125 µg per ml Triazine 12 and 12.5 mg sodium borohydride at pH 7.5 for a 10 min reaction time at room temperature (25 °C).^34–37^

### 2.7. Biolocalization

**Fig. 11:**
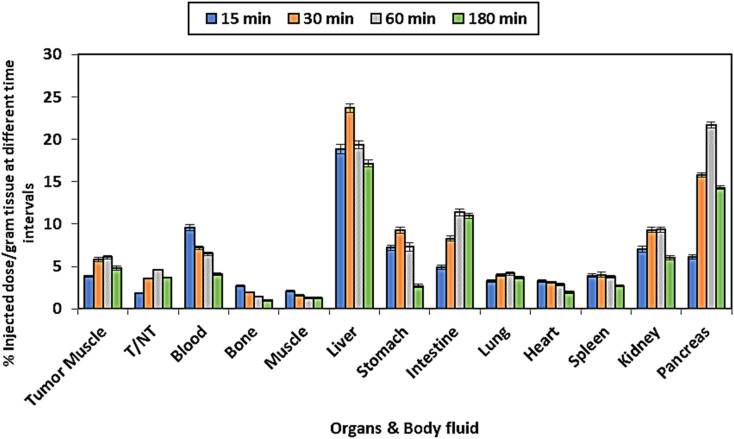
Uptake in organs as % injected dose per gram tissue at different time intervals, expressed as X ± S.D.

As a critical strategy for assessing the pharmacokinetic properties linked to newly developed therapeutic vectors, *in vivo* distribution studies play a leading role. Screening the biolocalization of [^99m^Tc]-Triazine 12, to be developed as a tumor targeting agent, is essential for gaining a comprehensive understanding of its potential. According to the docking and modeling studies, Triazine 12 was chosen to investigate the biodistribution pattern of [^99m^Tc]-Triazine 12. The bio-localization assessment was conducted 0.25, 0.5, 1, and 3 hours post-injection, with results reported as the percentage of the administered dose per gram of tissue. Accordingly, four distinct experimental groups of mice were utilized. After injecting [^99m^Tc]-Triazine 12 intravenously into the mice's tail vein, the in vivo-distributed profile revealed the pattern of the optimal bio-distribution seen with the functionalized anticancer agents. It was observed that most prominent organs exhibited a minimized-radioactive accumulation, with the shedding blood pool showing a criterion of radioactivity retention between 9.59 ± 0.34% ID g^−1^ at 0.25 hours and 4.1 ± 0.16% ID g^−1^ at 3 hours post-injection, as shown in the biodistribution (Fig. 11). Additionally, the liver and spleen, which are part of the reticuloendothelial system in addition to the pancreas, showed the highest uptake (19.37 ± 0.47%, 3.81 ± 0.15%, and 21.7 ± 0.35% ID g^−1^, respectively) 1-hour post-injection due to their leaky vasculature nature. At 30 minutes post-injection, a greater renal and intestinal uptake was seen, indicating significant kidney and hepatobiliary excretion.

In tumor-bearing mice, the *in vivo* distribution indicated an increasing accumulation associated with tumor tissues, reaching a peak uptake of 6.09 ± 0.21% ID g^−1^ 1-hour post-injection, followed by a gradual decrease to a minimum value. Notably, our findings suggest that Triazine 12 can be used as a drug delivery vehicle for transporting ^99m^Tc to tumor tissues. Furthermore, the antitumor activity is evaluated through the implementation of intermittent ratios (T/NT), calculated by comparing the uptake of tumor with the discarded organ (contralateral muscle). During the experiment, data consistently displayed and rendered positive T/NT assessments, reaching a peak of 4.65 1-hour post-injection (Fig. 11). This ratio is higher than that of previously found tumoral agents such as ^99m^Tc (CO)_3_-labeled chlorambucil analog (3.2 at 3 h) and [^99m^Tc]-sunitinib (3 at 1 h). Therefore, [^99m^Tc]-Triazine 12 may serve as a platform for therapeutic monitoring and diagnostic applications. All of the aforementioned findings show that [^99m^Tc]-Triazine 12 has high tissue selectivity for tumor cells and may be used as a novel, potentially effective tumor targeting agent. The [^99m^Tc]-Triazine 12 showed AMPK receptor targeting, evidenced by the highest uptake of AMP receptor-rich organs such as liver, pancreas, and spleen (Table 7).^78,79^ Also, Tc-Triazine 12 showed selective uptake in the negatively charged acidic organs such as the stomach. This uptake may explain the selective targeting towards the acidic environment of hypoxic solid tumors. Based on AMPK receptor selectivity and acidic media affinity, [^99m^Tc]-Triazine 12 could be a promising diagnostic vector for pancreatic and hepatic tumors, which also may highlight the possible role of Triazine 12, as a member of the condensed benzotriazinones, in serving as an antitumor drug for AMPK-rich organs.^37–42^

**Table 7:** The docking study of AMP-activated protein kinase (AMPK) for Triazine 12

PDB ID						
	2OOX		4CFF		4CFE	
	Ref. 78		Ref. 79		Ref. 79 18-27974-1	
	Score (kcal mol^−1^)	RMSD	Score (kcal mol^−1^)	RMSD	Score (kcal mol^−1^)	RMSD
Ref.	AMP	1.34895563	CIV	1.70577681	STU	1.54310167
12	−8.91851139		−5.62168646		−9.48375511	
	−7.06010866	1.62943828	−5.7900629	1.00726855	−7.16532516	1.91744721

## 3. Experimental

### 3.1. Chemicals

All chemicals and their reagents were obtained from commercial vendors and used without further purification. The thin layer chromatography observations were accomplished using percolated silica gel plates (Merk, art. 5715) and analyzed under UV light (254 nm). Both the spectra ^1^H NMR and ^13^C NMR were recorded on Bruker Avance 300 and 500 using NMR solvent DMSO-d_6_ and are reported as follows: chemical shift *δ* (ppm), multiplicity (s = singlet, d = doublet, dd = doublet of doublets, t = triplet, q = quartet, quint = quintet, sex = sextet, m = multiplet, b = broad). Electron spray ionization technique of LCMS analysis was implemented with an Agilent Technology 6130 quadrupole instrument.

#### 3.1.1 2-((1-Cyano-2-ethoxy-2-oxoethyl)diazenyl)benzoic acid (2)

To an ice-cold solution of 2-aminobenzoic acid (1.37 g, 0.01 mol) in a concentrated hydrochloric acid (2 ml) at 0–5 °C, a cold solution of NaNO_2_ (0.82 g, 0.01 mol) in 10 ml H_2_O was added (drop by drop) with vigorous stirring for fifteen minutes. Subsequently, the cold 2-(chlorodiazenyl) benzoic acid diazonium solution 1 was added to ethyl cyanoacetate in ethanol in the presence of sodium acetate at 0–5 °C. The reaction mixture was stirred for an additional hour; furthermore, the obtainable solid was collected and filtered. Accordingly, in each case, the crude product was recrystallized from EtOH–DMF mixture (2 : 1) and obtained as pale-yellow crystals (2.19 g, 79%) m. p.: 209–211 °C; FT-IR (ν max, cm^−1^): 1737 and 1669(CO), 2206 (C≡N nitrile). ^1^H NMR (500 MHz, DMSO-d_6_) *δ* = 1.20–1.28 (t, *J* = 2.60 Hz, 3H, O=C–O–CH_2_ CH_3_), 2.47 (s, 1H, CH CN), 4.26–4.30 (q, *J* = 1.35 Hz, 2H, O=C–O–CH_2_ CH_3_), 7.20–7.97 (m, 4H, Ar–H, s), 13.03 (s, 1H, OH). Anal. calcd for C_12_H_11_N_3_O_4_: C, 51.17; H, 4.24; N, 16.09. Found: C, 51.20; H, 4.19; N, 16.15.

#### 3.1.2 2-Hydroxy-10-oxo-5,10-dihydro-3*H*-benzo[*d*][1,2,3]triazolo[2,1-*a*][1,2,3]triazine-3-carbonitrile (4)

Compound 2 (2.77 g, 0.01 mol) and a solution of hydrazine (0.32 ml, 0.01 mol) were refluxed in *n*-butanol (10 ml) for 6 hours. The mixture was concentrated then filtered to generate red crystals of 4, (1.67 g, 73%) m. p.: >300 °C; FT-IR (ν max, cm^−1^): 3444 (OH), 3239 (NH), 2225 (C≡N nitrile), 1670, (CO). ^1^H NMR (500 MHz, DMSO-d_6_) *δ* = 3.52 (s, 1H, CH–CN), 8.10–7.51 (m, 4H, Ar–H, s), 10.47 (s, 1H, cyclic NH), 14.24 (s, 1H, OH) (*λ*_max_ = 448.8 nm, 8133). Anal. calcd for C_10_H_7_N_5_O_2_: C, 52.40; H, 3.08; N, 30.56. Found: C, 52.38; H, 3.12; N, 30.50.

#### 3.1.3 Ethyl 2-cyano-2-(4-oxo-3-(phenylamino)-3,4-dihydrobenzo[*d*][1,2,3] triazin-2(1*H*)-yl)acetate (5)

A mixture of 2 (2.77 g, 0.01 mol) and phenyl hydrazine (0.99 ml, 0.01 mol) was refluxed in *n*-butanol (10 ml) for 7 h. The obtainable precipitate upon concentration and cooling was filtered to give 5, which was crystalized in a EtOH–DMF mixture (2 : 1) to give the yellow powder of 5, (2.56 g, 73%) m. p.: 228–230 °C; FT-IR (ν max, cm^−1^): 3247 (NH), 2233 (CN), 1704 (CO), and 1602 (C=O). ^1^H NMR (500 MHz, DMSO-d_6_) *δ* = 1.28 (t, *J* = 2.65 Hz, 3H, OC = O CH_2_CH_3_), 4.29–4.26 (q, *J* = 1.30 Hz, 2H, O=C–O CH_2_CH_3_), 4.9 (s, 1H, CH), 7.94–7.63 (m, 9H, Ar–H,s), 13.07 (s, 1H, *vic*-triazine NH), 14.53 (s, 1H, NHPh) (*λ*_max_ = 469.6 nm, 2086). Anal. calcd for C_18_H_17_N_5_O_3_: C, 61.53; H, 4.88; N, 19.93. Found: C, 61.30; H, 4.60; N, 19.90.

#### 3.1.4 Ethyl 2-cyano-2-(3-cyano-4-oxo-3,4-dihydrobenzo[*d*][1,2,3]triazin-2(1*H*)-yl)acetate (6)

To a solution of the compound 2 (2.77 g, 0.01 mol) in *n*-butanol (10 ml), guanidine (0.59 g, 0.01 mol) was added and refluxed for 6 hours. The obtainable crystals, upon cooling, were recrystallized from the EtOH/DMF mixture (2 : 1). Yellow powder (2.07 g, 69%); m. p.: >300 °C; FT-IR (ν max, cm^−1^): 3448 (br, NH), 2210 (CN nitrile), 1704, and 1592 (CO). ^1^H NMR (500 MHz, DMSO-d_6_) *δ* = 1.34 (t, *J* = 2.60 Hz, 3H, O=C–O–CH_2_CH_3_), 4.14–4.12 (q, *J* = 1.36 Hz, 2H, O=C–O–CH_2_CH_3_), 4.76 (s, 1H, CH), 7.89–7.02 (m, 4H, Ar–H, s), 15.40 (s, 1H, triazine NH). Anal. calcd for C_13_H_11_N_5_O_3_: C, 54.74; H, 3.89; N, 24.55. Found: C, 54.69; H, 3.82; N, 24.67.

#### 3.1.5 Ethyl 2-(3-carbamoyl-4-oxo-3,4-dihydrobenzo[*d*][1,2,3]triazin-2(1*H*)-yl)-2-cyanoacetate (7)

A mixture of 2 (2.77 g, 0.01 mol) and urea and/or thiourea (0.60 g and/or 0.76 g, 0.01 mol) was refluxed in 10 ml *n*-butanol for 6 hours. The reaction mixture was cooled to room temperature, and the precipitation, filtration, and recrystallization from EtOH/DMF mixture (2 : 1) produced 7 as green crystals (1.90 g, 63%); m. p.: 254–256 °C; FT-IR (ν max, cm^−1^): 3463 (NH), 2229 (CN nitrile), 1690, and 1647 (CO), 1600 (C=C). ^1^H NMR (500 MHz, DMSO-d_6_) *δ* = 1.27–1.23 (t, *J* = 2.70 Hz 3H, CO_2_CH_2_CH_3_), 4.28–4.18 (q, *J* = 1.65 Hz 2H, CH_2_CH_3_), 4.92 (s, 1H, CH),6.78 (s, 2H, NH_2_), 7.91–7.00 (m, 4H, Ar–H, s), 7.72(s, 1H, triazine NH). ^13^C NMR (125 MHz, DMSO-d_6_) *δ* = 19.16, 6.69, 62.49, 103.64, 115.41, 123.58131.72, 131.91, 133.68, 143.24, 148.60, 160.17, 169.57 (*λ*_max_ = 462 nm, 1142). Anal. calcd for C_13_H_13_N_5_O_4_: C, 51.49; H, 4.32; N, 23.09. Found: C, 51.40; H, 4.30; N, 23.20.

#### 3.1.6 Ethyl 2-cyano-2-(3-(2-cyanoacetamido)-4-oxo-3,4-dihydrobenzo[*d*][1,2,3]triazin-2(1*H*)-yl)acetate (8)

A mixture of 2 (2.77 g, 0.01 mol) and cyanoacetohydrazide (0.99 g, 0.01 mol) was refluxed in 10 ml *n*-butanol for 6 hours. The reaction mixture was cooled to room temperature and precipitated, filtered, and recrystallized from an EtOH/DMF mixture (2 : 1) to produce 8 as a yellow powder (2.36 g, 67%) m. p.: 280–282 °C; FT-IR (ν max, cm^−1^): 3486, 3216, 3166 (NH), 2210 (CN nitrile), 1739, and 1697 (CO), 1596 (C=C). ^1^H NMR (500 MHz, DMSO-d_6_) *δ* = 1.22–1.34 (t, *J* = 2.65 Hz 3H, CH_2_CH_3_), 3.05 (s, 1H, CH), 4.16–4.19 (q, *J* = 1.65 Hz 2H, O=C–OCH_2_CH_3_), 4.20 (s, 1H, CH), 5.84 (s, 1H, cyclic NH), 7.01–7.04 (t, *J* = 8.00 Hz,1H, Ar–H), 7.42–7.46 (t, *J* = 8.00 Hz,1H, Ar–H), 7.56–7.58 (d, *J* = 8.00 Hz,1H, Ar–H), 7.89–7.91 (d, *J* = 8.00 Hz,1H, Ar–H), 7.96 (s, 1H, NH), ^13^C NMR (125 MHz, DMSO-*d*_6_) *δ* = 14.14, 19.23, 60.43, 61.77, 115.00, 115.14, 120.85, 123.26, 124.48, 131.66, 132.11, 133.15, 150.73, 163.74, 169.30 (*λ*_max_ = 470.6 nm, 1749). Anal. calcd for C_15_H_14_N_6_O_4_: C, 52.63; H, 4.12; N, 24.55. Found: C, 52.60; H, 4.09; N, 24.82.

#### 3.1.7 Ethyl 2-(3-benzyl-4-oxo-3,4-dihydrobenzo[*d*][1,2,3]triazin-2(1*H*)-yl)-2-cyanoacetate (9)

The solution of compound 2 (2.77 g, 0.01 mol) and benzyl amine (1.07 g, 0.01 mol) was refluxed in *n*-butanol 10 ml for 6 hours. The obtainable crystals, upon cooling, were recrystallized from EtOH/DMF mixture (2 : 1) to produce 9 as a green powder (2.73 g, 78%) m. p.: 172–174 °C; FT-IR (ν max, cm^−1^): 3432 (NH), 2210 (CN nitrile), 1739, and 1693 (CO), 1591 (C=C). ^1^H NMR (500 MHz, DMSO-d_6_) *δ* = 1.5–0.90 (t, *J* = 2.00 Hz 3H, CH_3_), 2.46 (s, 1H CH), 4.14–4.02 (q, *J* = 1.70 Hz 2H, O=C–OCH_2_CH_3_), 4.46 (br 3H, NH + CH_2_Ph), 7.89–7.35 (m, 9H, Ar–H, s).^13^C NMR (125 MHz, DMSO-d_6_) *δ* = 14.50, 19.24, 31.03, 64.14, 114.97, 115.08, 123.26, 128.93, 129.13, 129.34, 1331.65, 133.15, 134.77, 150.66, 163.70, 169.27 (*λ*_max_ = 465.4 nm, 4065). Anal. calcd for C_19_H_18_N_4_O_3_: C, 65.13; H, 5.18; N, 15.99. Found: C, 65.09; H, 5.20; N, 15.91.

#### 3.1.8 Ethyl 2-(3-(2-aminophenyl)-4-oxo-3,4-dihydrobenzo[*d*][1,2,3]triazin-2(1*H*)-yl)-2-cyanoacetate (10)

A solution of target 2 (2.77 g, 0.01 mol) and *o*-phenylenediamine (1.0814 g, 0.01 mol) was heated under reflux in *n*-butanol 10 ml for 6 hours. The obtainable crystals were recrystallized from an EtOH/DMF mixture (2 : 1). Greenish-brown crystals (2.17 g, 62%), m. p.: 182–184 °C; FT-IR (ν max, cm^−1^): 3419–3359 (NH, NH_2_), 2225 (CN nitrile), 1735, and 1689 (CO), 1598 (C=C). ^1^H NMR (500 MHz, DMSO-d_6_) *δ* = 1.36–1.26 (t, *J* = 2.70 Hz 3H, O=C–OCH_2_CH_3_), 2.46 (s, 1H CH), 3.35 (s, 2H NH_2_), 4.30–4.26 (q, *J* = 1.65 Hz 2H, O=C–OCH_2_CH_3_), 5.1 (s, 2H, NH_2_),7.91–6.46 (m, 9H, Ar–H, s), 14.1 (s, 1H, NH cyclic). ^13^C NMR (125 MHz, DMSO-d_6_) *δ* = 14.86, 61.03, 62.53, 115.23, 119.01, 123.26, 124.92, 131.73, 131.93, 133.86, 143.18, 148.70.162.72, 169.44 (*λ*_max_ = 522.5 nm, 6478). Anal. calcd for C_18_H_17_N_5_O_3_: C, 61.53; H, 4.88; N, 19.93. Found: C, 61.50; H, 4.70; N, 19.90.

#### 3.1.9 *N*-(2-(1-Cyano-2-ethoxy-2-oxoethyl)-4-oxo-1,4-dihydrobenzo[*d*][1,2,3]triazin-3(2*H*)-yl)carbamimidothioic acid (12)

A mixture of 2 (2.77 g, 0.01 mol) and thiosemicarbazide (0.91 g, 0.01 mol) was refluxed in *n*-butanol 10 ml for 6 h. The reaction mixture was cooled to room temperature, filtered and recrystallized from EtOH/DMF mixture (2 : 1). Yellow powder (2.75 g, 68%); m. p.: 288–290 °C; FT-IR (ν max, cm^−1^): 3405 (NH), 2151 (CN nitrile), 1681 (CO), 1612 (C=C), 1276 (C=S). ^1^H NMR (500 MHz, DMSO-d_6_) *δ* = 1.22–1.24 (t, *J* = 1.77 Hz 3H, O=C–OCH_2_CH_3_), 1.91 (s, 1H, SH), 4.17–4.21 (q, *J* = 1.72 Hz 2H, O=C–OCH_2_CH_3_), 4.99 (s, 1H CH), 6.79 (s, 1H, NH), 7.04–7.07 (t, 1H, *J* = 8.00, Ar–H), 7.21 (s, 1H, NH), 7.45–7.48(t, 1H, *J* = 7.01, Ar–H), 7.57–7.59 (d, 1H, Ar–H), 7.89–7.91 (d, 1H, Ar–H), 12.28 (s, 1H, NH), 12.75 (s, 1H, NH). Anal. calcd for C_13_H_14_N_6_O_3_S: C, 46.70; H, 4.22; N, 25.14. Found: C, 46.60; H, 4.16; N, 25.45.

#### 3.1.10 Cinnolin-4(3*H*)-one (14)

A mixture of 2 (2.77 g, 0.01 mol) and semicarbazide (1.11 g, 0.01 mol) was refluxed in *n*-butanol 10 ml for 8 h. The reaction mixture was cooled, filtered, and the obtained product was recrystallized from an EtOH/DMF mixture (2 : 1). Yellow powder; (1.00 g, 72%) m. p.: 238.5–239.5 °C; FT-IR (ν max, cm^−1^): 1681 (CO). ^1^H NMR (500 MHz, DMSO-d_6_) *δ* = 5.99 (s, 2H, CH_2_), 7.63–7.57 (m, 4H, Ar–H, s). Anal. calcd for C_8_H_6_N_2_O: C, 65.75; H, 4.14; N, 19.17. Found: C, 65.70; H, 4.10; N, 19.20.

### 3.2. Molecular docking

As per the literature, a simulation of molecular docking is achieved utilizing the Molecular Operating Environment (MOE) software 2002.^12,43^ A force field called MMFF94x was used to decrease energy after ligands were constructed using the MOE builder, which was 3D protonated and partially charged. From the RCSB-Protein Data Bank, the protein targets were sourced. 5V67, 1RR8, 3IG7, 4ASD, 3FC4, 1QZR, and 1YET^44–46^ binding sites were used, along with blank and volasertib. Automated 3D protonation of atoms and connections followed by potential fixing was the typical preparation protocol used to make the proteins. Ultimately, the MOE alpha site finder was utilized to identify the active sites, and fake atoms would be created as alpha spheres to symbolize the hydrophobic and polar elements of the active sites of the receptors (*i.e.*, 5V67, 1RR8, 3IG7, 4ASD, 3FC4, 1QZR, and 1YET). A procedure for induced-fit docking in MOE was applied. As part of a typical validation approach, the native ligand was re-docked to the active sites of 5V67, 1RR8, 3IG7, 4ASD, 3FC4, 1QZR, and 1YET,^47–50^ with a cutoff root-mean-square deviation (RMSD) value of less than 2.0. Using the London dG scoring function as the first scoring function and the triangle matcher technique, the interaction of ligands at the active sites was investigated. Following the use of thirty poses, five were kept. The second scoring function is the force field-based GBVI/WSA dG scoring function. Records were made of conformations with a lower RMSD and a higher S-value.^48,51–57^

### 3.3. Antioxidant capacity determination

The 2,2-diphenyl-1-picryl-hydrazyl-hydrate (DPPH) free radical assay is an antioxidant assay based on electron transfer and produces a violet solution in ethanol. The DPPH assay is an easy and rapid method to estimate the antioxidant activity of a certain product by spectrophotometry. The DPPH radical is stable at room temperature and can be reduced by an antioxidant molecule, resulting in a colorless ethanol solution.

The antioxidant activity percentage (AA%) of a substance is the measurement of the DPPH radical scavenging activity (RSC) according to methods described by Santos *et al.*,^58^ with some modifications to methods they had described as follows: DPPH ethanolic solution (0.1 mM) was prepared and served as the stock solution. The control sample was 4 ml DPPH solution, while the test sample was 0.2 ml sample added to 3.8 ml DPPH solution. All samples were then kept at room temperature in the dark for 30 min before their absorbance were measured at 517 nm by U. V. spectrophotometer. The following equation estimated the percentages of radical scavenging activity (%RSC):^58,59^
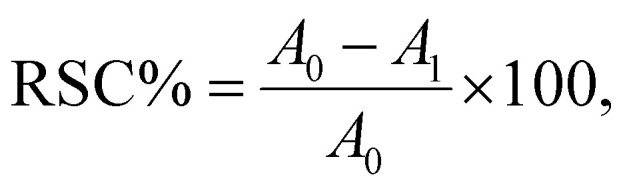
where *A*_0_ the absorbance of the control sample and *A*_1_ is the absorbance of the test sample.

### 3.4. *In vitro* evaluation of the cytotoxic activity of Triazine 12

Triazine 12 was assessed *in vitro*, implementing viability assays (MTT assay). Its cytotoxicity against human breast cancer cell line (MCF-7) and human hepatocellular carcinoma cell line (HepG-2) was tested at the Regional Centre for Microbiology and Biotechnology, Cairo, Egypt, according to Mosmann's protocol.^60^ All cell lines used were provided from the American Type Culture Collection (ATCC, Rockville, MD). These cells were grown in RPMI-1640 medium complemented with 10% inactivated fetal calf serum and 50 µg per ml gentamycin. These cells were cultured at 37 °C in a moist atmosphere with 5% CO_2_ and subcultured twice to three times a week.

For the assay, suspensions of cell lines in medium were placed at a concentration of 5 × 10^4^ cells per well in a Corning® 96-well tissue culture plate, which was incubated for 24 h. Triazine 12 was then added to 96-well plates (three replicates). Six wells with media or 0.5% DMSO were used for each 96-well plate as a control. After incubation for 24 h, the viable cell ratios were calculated and determined by the MTT test.^60^

### 3.5. *In vitro* biological evaluation of normal human lung fibroblast cells for MRC-5

Upon characterization, the produced Triazine 12 was evaluated *in vitro* using viability assays (MTT assay). The cytotoxicity of Triazine 12 was tested against normal human lung fibroblast cells (MRC-5) at the Regional Centre for Microbiology and Biotechnology, Cairo, Egypt, according to Brayner's protocol.^61^

All cell lines used were provided from the American Type Culture Collection (ATCC, Rockville, MD). The cells were grown in RPMI-1640 medium supplemented with 50 g per ml gentamycin and 10% inactivated fetal calf serum purchased from Lonza (Belgium). The cells were subcultured every two days and incubated in 5% CO_2_ at 37 °C.^62^

### 3.6. *In vitro* evaluation of the cytotoxic mechanism of action of Triazine 12

In order to study the possible mechanism of action on the HepG2 cell lines, the levels of apoptotic markers caspase-3 and Bax, and that of the anti-apoptotic marker Bcl-2, were analyzed as using ELISA colorimetric kits (R&D Systems, USA) at the Regional Centre for Microbiology and Biotechnology, Cairo, Egypt. HepG2 cells were seeded at a density of 5 × 10^5^ cells per ml into 6-well plates a day before the experiment. After the formation of a complete cell monolayer in each well of the plate, Triazine 12 was dispensed into the 6-well tissue culture plates at IC_50_ concentration. Each treatment was performed in triplicate.^63,64^

### 3.7. Radiolabeling of Triazine 12 with ^99m^Tc

Briefly, 12.5 mg NaBH_4_ was added to a pre-cooled penicillin vial, followed by the addition of 100 µl of Na^99m^TcO_4_ (200 MBq). The vial was shaken well until all the borohydride was dissolved, and then 125 µg ml^−1^ of Triazine 12 solution was quickly added to the mixture. The reaction was optimized for variable reaction factors, including the substrate Triazine 12 concentration (50–200 µg ml^−1^), the reducing agent (borohydride) amount (2.5–25 mg), pH values,^5–12^ reaction time^1–60^ and temperature (8–75 °C) to obtain maximum radiolabeling yield of [^99m^Tc]-Triazine 12.^65,66^

The radiochemical yield (RCY%) of [^99m^Tc]-Triazine12 was evaluated using the ascending paper chromatography technique. Two strips of Whatman No. 1 chromatography paper (13 cm long and 1 cm wide) were marked at a distance of 2 cm from the lower end and lined into sections 1 cm each up to 10 cm. Spots of one or two drops from the mixture were placed using a hypodermic syringe, then the strips were put in a jar containing a developing solution. One of them was acetone, and the other was a mixture of water/ethanol/ammonia at a ratio of 5 : 2 : 1. After complete development, the strips were dried and cut into 1 cm sections, which then were counted by NaI (Tl) γ-ray scintillation counter (SPECTECH, ST450 SCA, USA) for measuring radioactivity.^67–69^

### 3.8. Tumor transplantation in mice

Ehrlich ascites carcinoma cells (EAC) are widely used as a model to study the biological behavior of malignant tumors and their interaction with drugs. These cells are expected to show effects at specific sites. The EAC line was maintained in female Swiss albino mice by injecting 2.5 × 10^6^ tumor cells intraperitoneally each week. The EAC cells were collected *via* needle aspiration under sterile conditions, and the ascitic fluid was diluted with sterile saline. A 0.1 ml solution containing 2.5 × 10^6^ cells was counted microscopically using a hemocytometer. Then, 0.2 ml of this solution was injected intramuscularly into the right leg muscle to induce a solid tumor model.^36,38^ Mice with a tumor diameter of 1 to 1.5 cm were chosen to proceed into the next step, while those with a diameter above or below this range were excluded.

### 3.9. Biodistribution of the labeled [^99m^Tc]-Triazine 12 in tumor-bearing mice

The institutional EAEA central committee approved all experimental protocols on animals for research ethics with ref. no. 233/2024, which states that all methods should be carried out in accordance with EU Directive 2010/63 guidelines and regulations. Moreover, all methods were reported in accordance with ARRIVE guidelines. The biolocalization assessment utilized 24 adult Swiss albino male mice in total, aged 7 weeks and weighing 25–30 g, which were randomly allocated by an independent technician from the animal facility staff into four groups according to specific time points post-injection (0.25, 0.5, 1, and 3 hours). They were obtained from the animal housing facility, Hot Labs center, EAEA (Inshas, Egypt). The facility maintained a temperature of 25 °C, relative humidity of 50%, and a constant 12-h light-dark cycle, and animals were allowed to access food and water *ad libitum.* The number of mice was calculated using G*Power 3.1.9.4 analysis software and statistical testing with ANOVA: fixed effects, omnibus, one-way.

Each group of six mice contained three normal mice plus three tumor-bearing mice. All mice were intravenously injected with 0.2 ml saline containing [^99m^Tc]-Triazine 12 with a radiation dose of approximately 5–10 kBq *via* the tail vein. To minimize bias, the dose injection procedure was performed by an independent technician from the animal facility staff. The mice were kept calm in metabolic cages before sacrifice at the post-injection time points. Mice have a recorded weight range of 27.5 to 33.4 g at the time of sacrifice. Then, they were sacrificed after anesthesia using ketamine/xylazine at 30/5 mg kg^−1^, followed by cervical dislocation to confirm death, and then dissection of various organs and tissues to be removed, weighed, and counted to assess their uptake of the radioactive probe. The results were calculated as a percentage of the injected dose (ID) per gram of tissue or organ in comparison to a standard containing 100% of the injected dose.^10^ Blood, bone, and muscle weights were estimated as 7%, 10%, and 40%, respectively, of the total body weight.^36,38,39,70–77^

## 4. Conclusions

This study shows the *de novo* synthesis of a new series of benzotriazinones, followed by full *in silico* docking studies. The most potent *in silico* molecule from the synthesized compounds, Triazine 12, showed significant antitumor activity, as demonstrated by docking studies. Subsequently, Triazine 12 was selected to further screen its biological activity *in vitro* and it showed promising *in vitro* antitumoral activity. Overall, further evaluation of Triazine 12 against normal fibroblast cells confirmed its low cytotoxicity, while the moderate antioxidant effect observed by the DPPH assay suggests an additional functional benefit that may enhance its potential as a safe and multifunctional anticancer candidate. In addition, efficient radiolabeling of Triazine 12 with [^99m^Tc] resulted in an impressive radiochemical yield of 95.4% ± 0.46% after the systematic optimization of key parameters, including minimization of the reducing agent amount, substrate amount, pH level, and reaction time. Biodistribution studies revealed the high affinity of [^99m^Tc]-Triazine 12 in tumor localization, highlighting its potential as a promising radiotracer for tumor imaging. Furthermore, Triazine 12 showed significant biochemical antitumor effects. Radiolabelled [^99m^Tc]-Triazine 12 showed high selectivity, confirming its value as a valuable tool for the precision imaging of solid tumors and possible treatment approaches.

## Author contributions

Wael Shehta contributed to conceptualization, investigation, methodology, chemical analysis, formal analysis, validation, resources, writing, reviewing, and editing the manuscript. Doaa A. Elsayed contributed to chemical analysis, formal analysis, resources, reviewing, editing the manuscript, *in silico* investigations (docking and modeling), writing validation, and drafting the original version of the paper. Fawzy Marzook was involved in biochemical *in vitro*, *in vivo* and radiolabeling studies, methodology, resources, validation, and drafting the original version of the paper. Mohammed G. Assy contributed to chemical analysis, formal analysis, validation, and resources. He also played a key role in writing, reviewing, and editing the manuscript. Mahmoud M. Sultan contributed to conceptualization, validation, synthesis, reviewing and editing the writing. S. El-Kalyoubi contributed to chemical analysis, validation, and resources, writing, reviewing, and editing the manuscript. M. Korany was involved in biochemical *in vitro*, *in vivo* and radiolabeling studies, methodology, resources, validation, and writing of the paper. Mohamed E. Abdu, Mohamed Abdel-Haleem and Mohamed Taha Yassin contributed to conceptualization, validation, synthesis, reviewing and editing the writing.

## Conflicts of interest

The authors state that they have neither known competing financial interests nor personal relationships that may appear to influence the reported work in this paper.

## Supplementary Material

RA-015-D5RA05853H-s001

RA-015-D5RA05853H-s002

## Data Availability

All data generated or analysed during this study are included in this published article [and its supplementary information (SI)]. Supplementary information is available. See DOI: https://doi.org/10.1039/d5ra05853h.
