# Supplementary material for: The novel synthesis of a condensed triazine via the heterocyclization of an azo derivative and its characterization, radiolabeling and bio-evaluation
Source: RSC Adv. 2025 Oct 21;15(47):39988–40005. doi: 10.1039/d5ra05853h (PMC12538673; doi:10.1039/d5ra05853h)
Supplement: RA-015-D5RA05853H-s002 [file RA-015-D5RA05853H-s002.pdf]

Supplementary Information (SI) for RSC Advances.
This journal is © The Royal Society of Chemistry 2025

SPECTRAL ANALYSIS

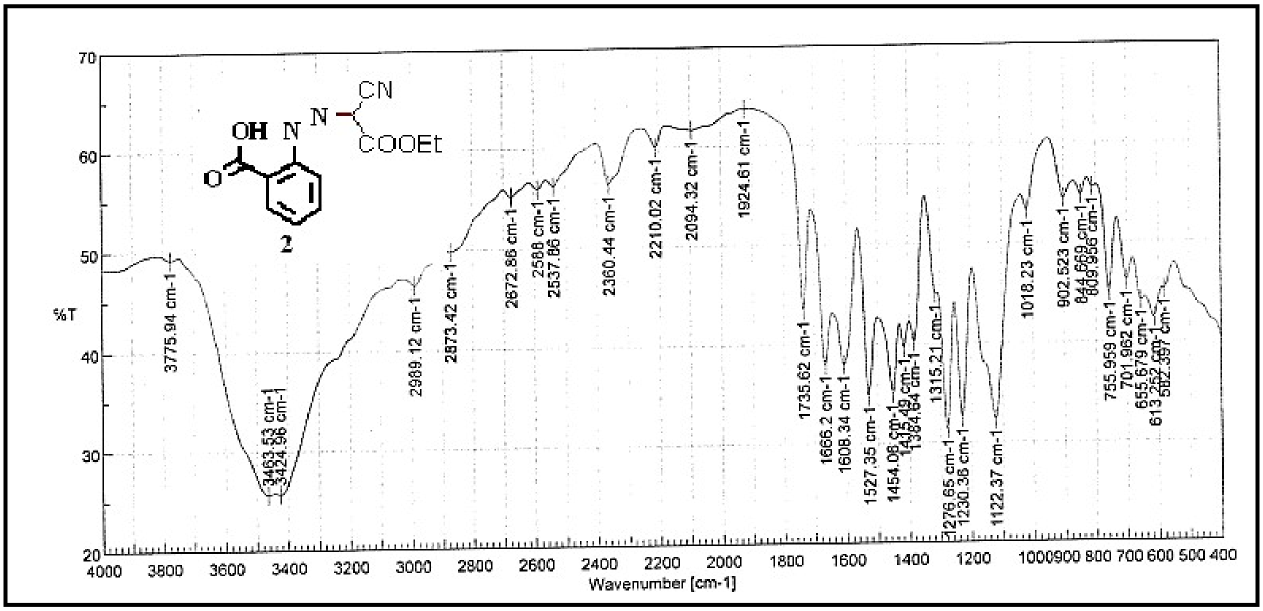

Fig. 1: **IR Spectrum of compound 2**

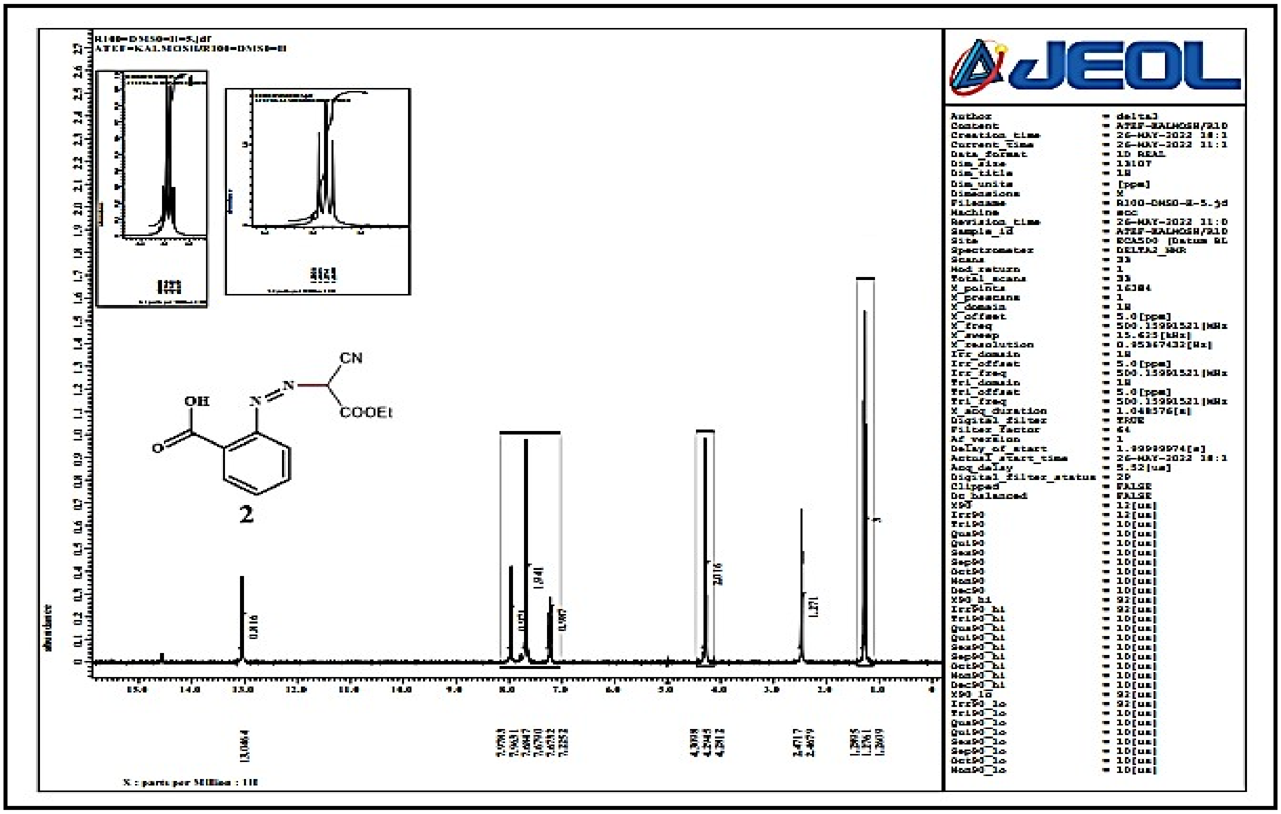

Fig. 2: **^{1}H-NMR Spectrum of compound 2**

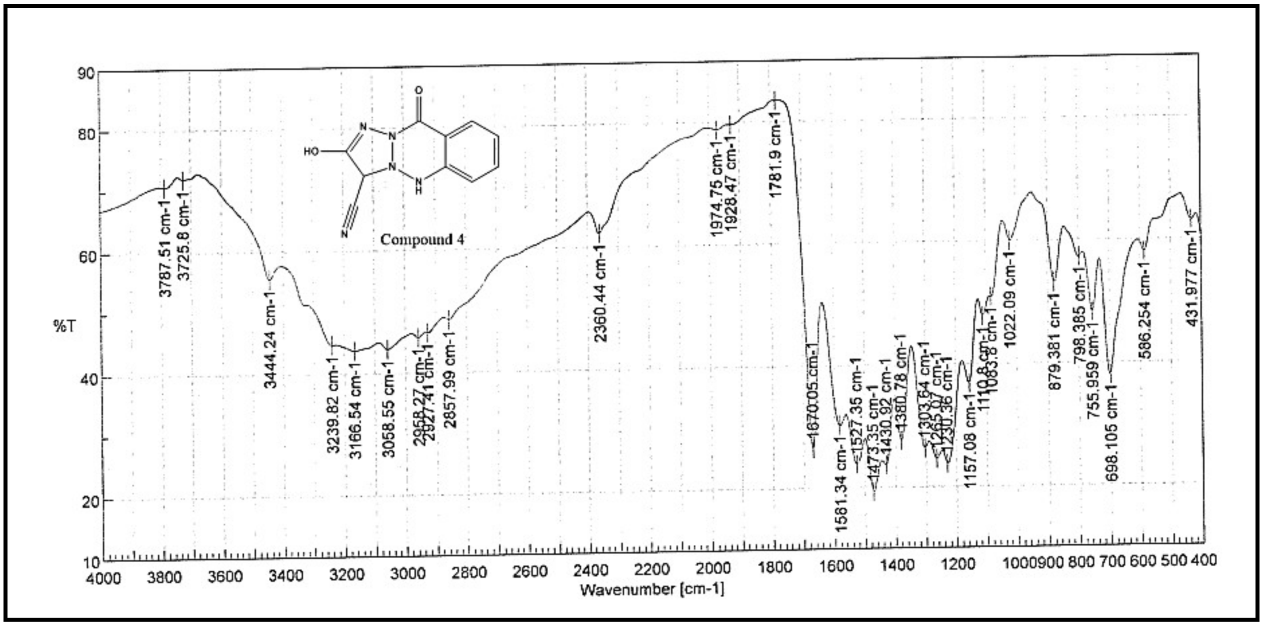

Fig. 3: **IR Spectrum of compound 4**

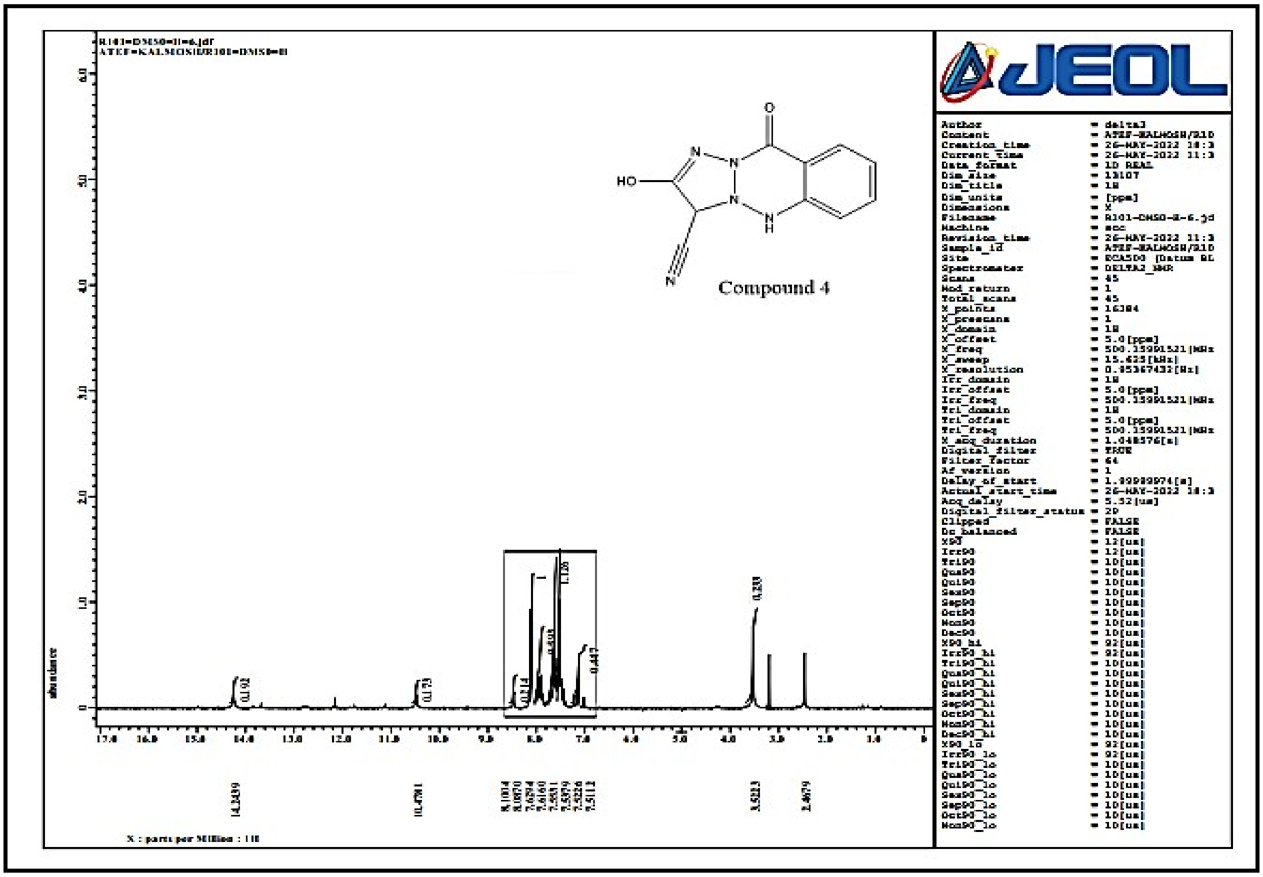

Fig. 4: **^{1}H-NMR Spectrum of compound 4**

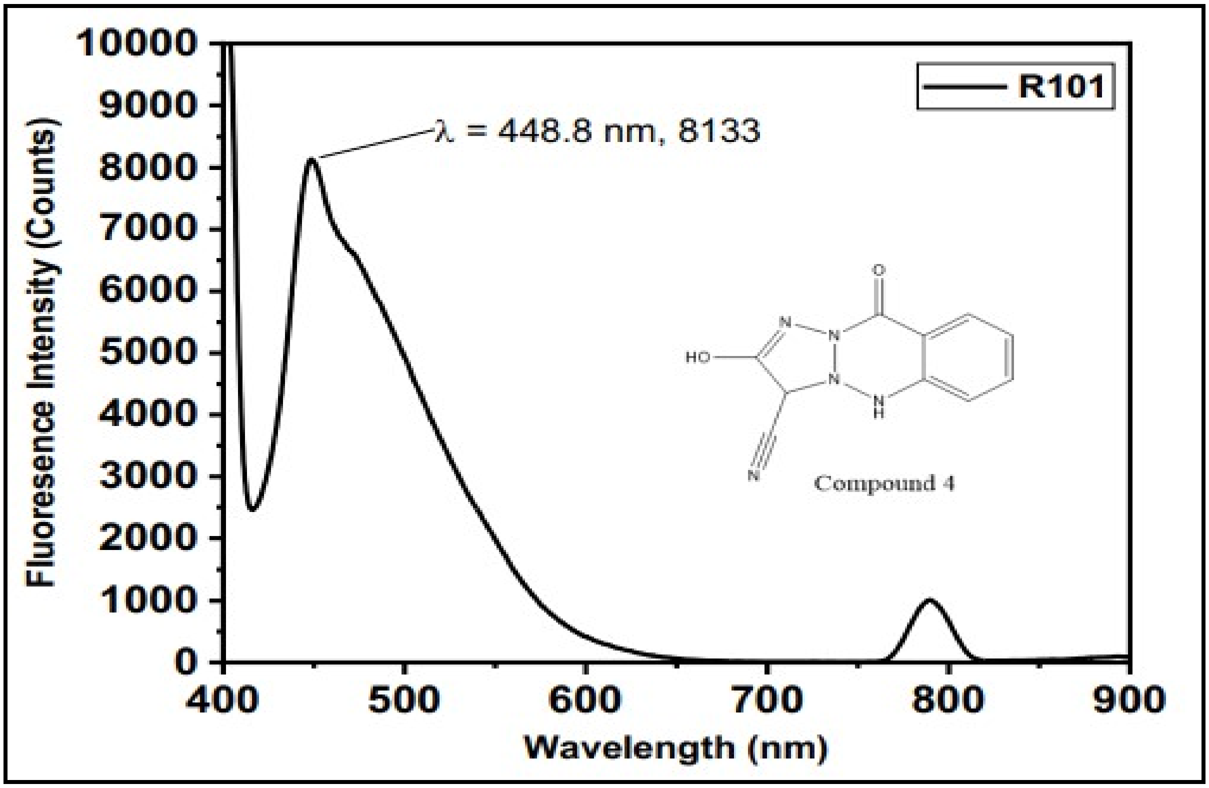

Fig. 5: **Fluorescence spectra of compound 4**

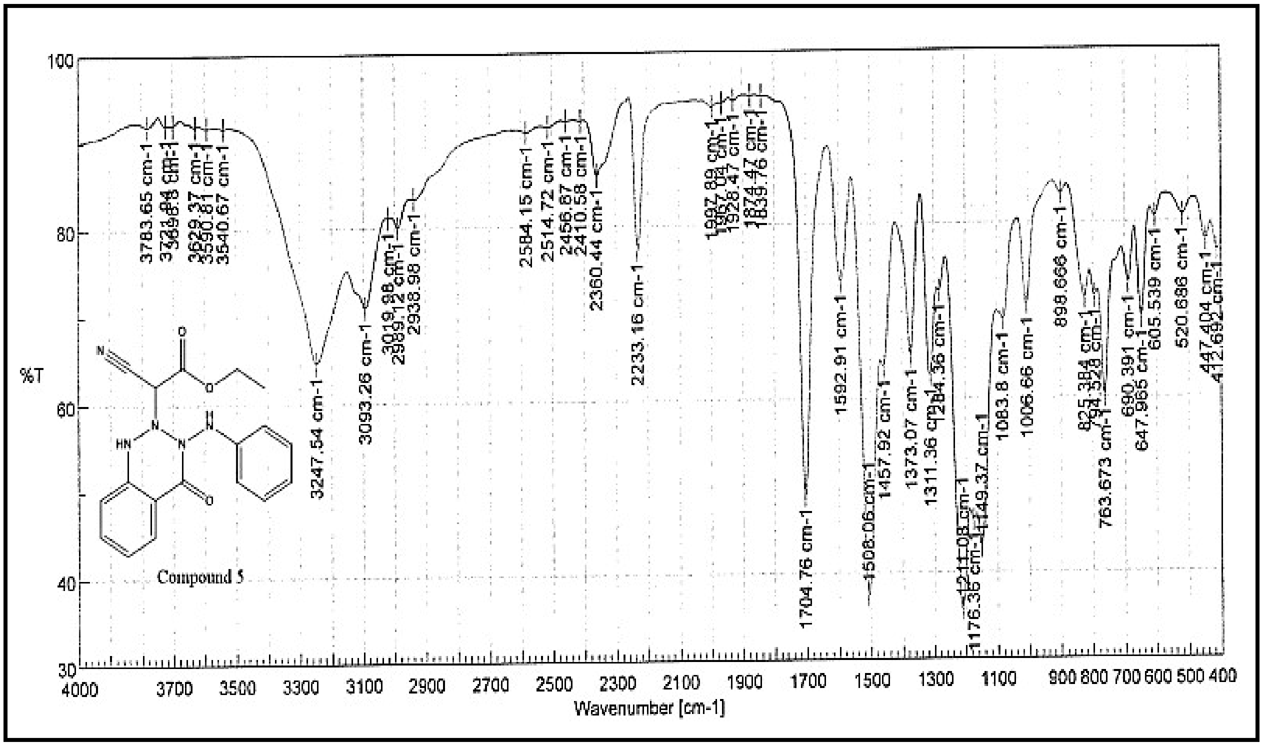

Fig. 6: **IR Spectrum of compound 5**

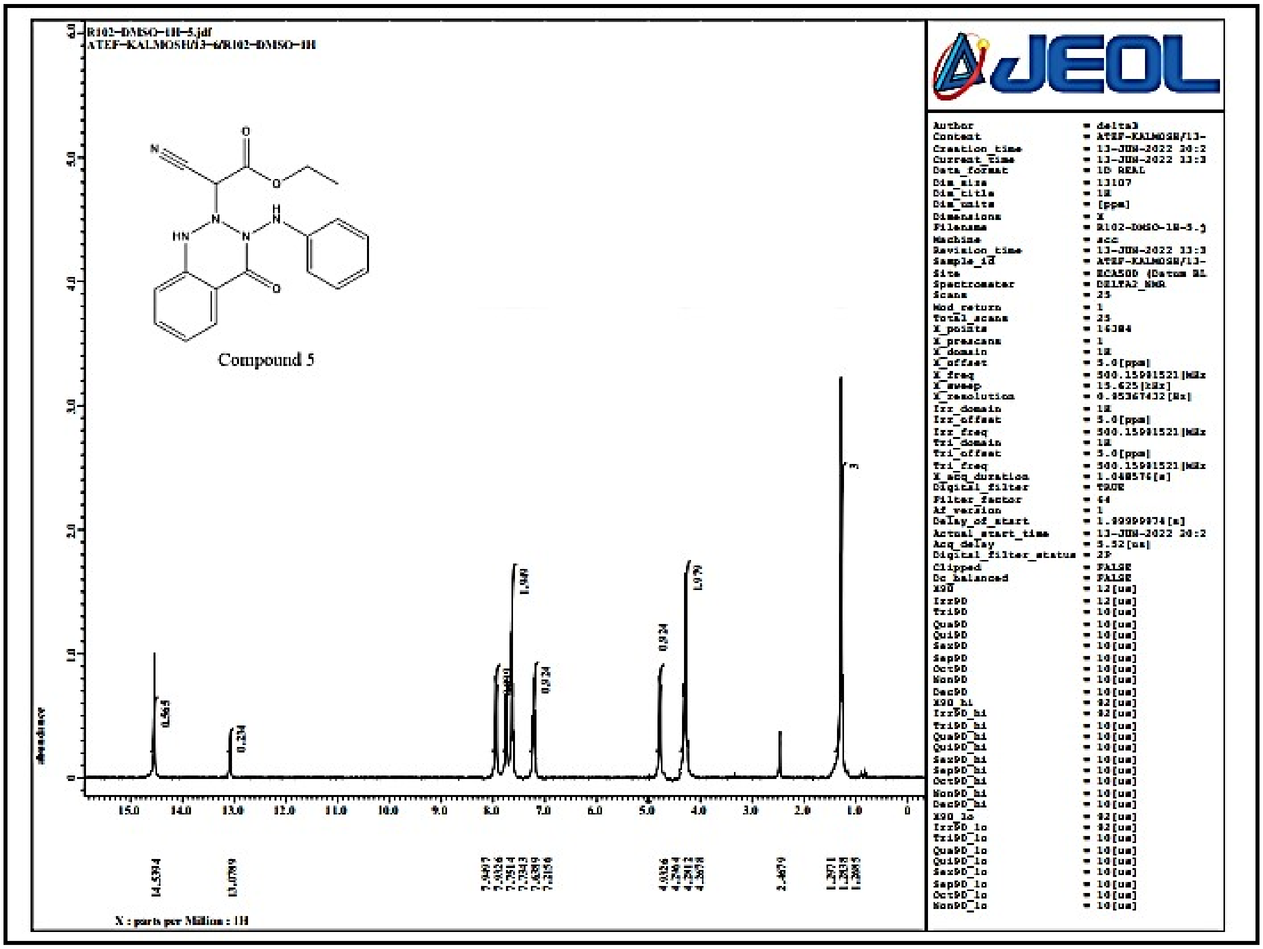

Fig. 7: **^{1}H-NMR Spectrum of compound 5**

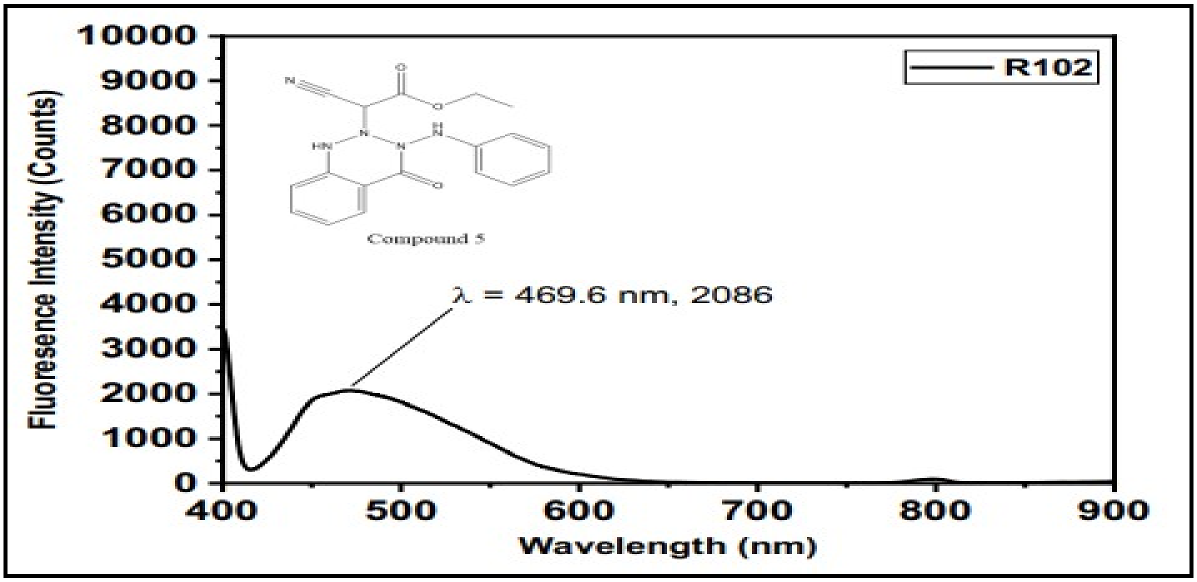

Fig. 8: **Fluorescence spectra of compound 5**

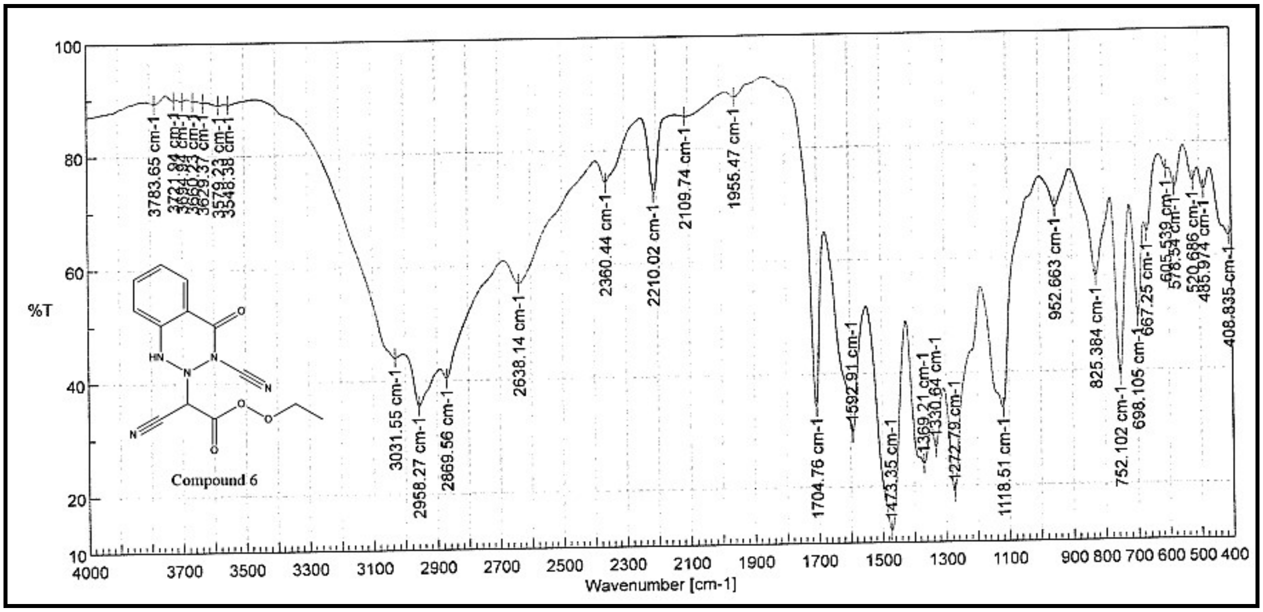

Fig. 9: **IR Spectrum of compound 6**

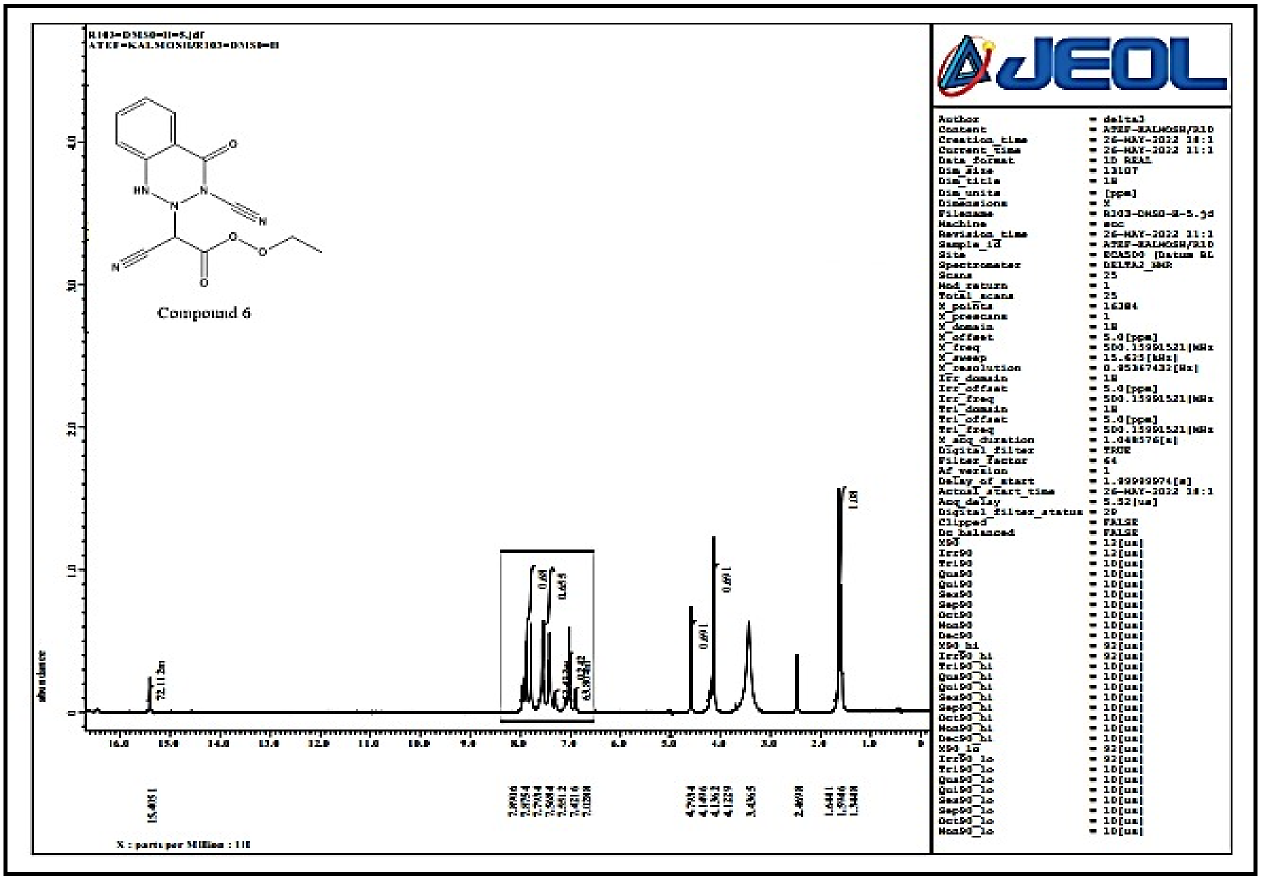

Fig. 10: **^{1}H-NMR Spectrum of compound 6**

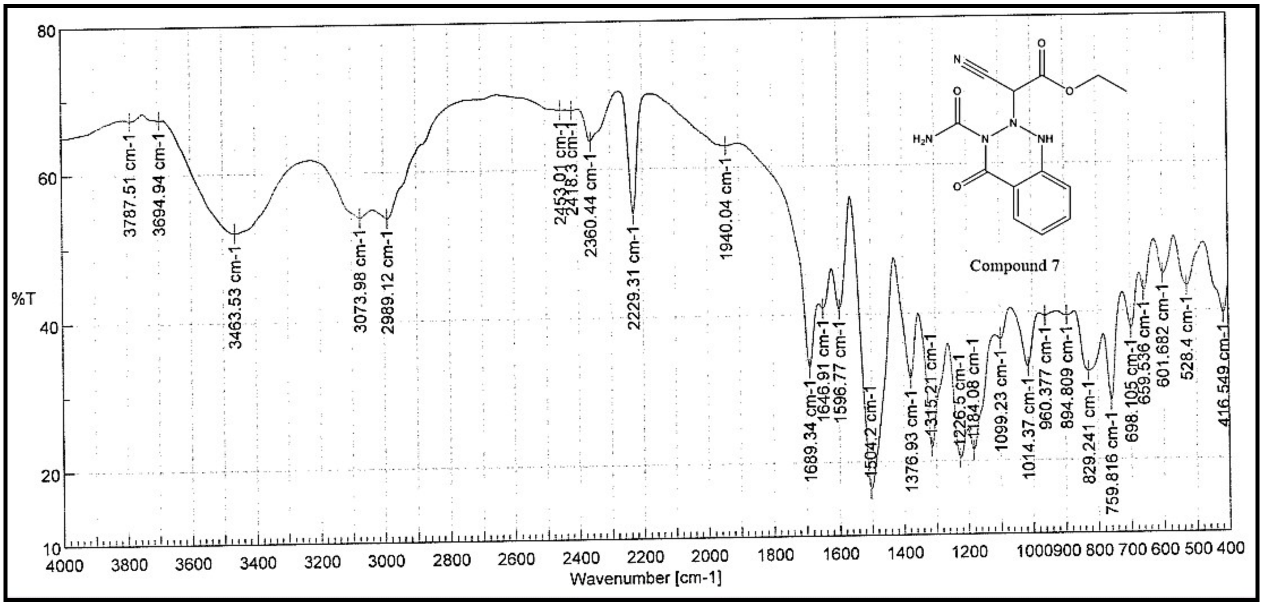

Fig. 11: **IR Spectrum of compound 7**

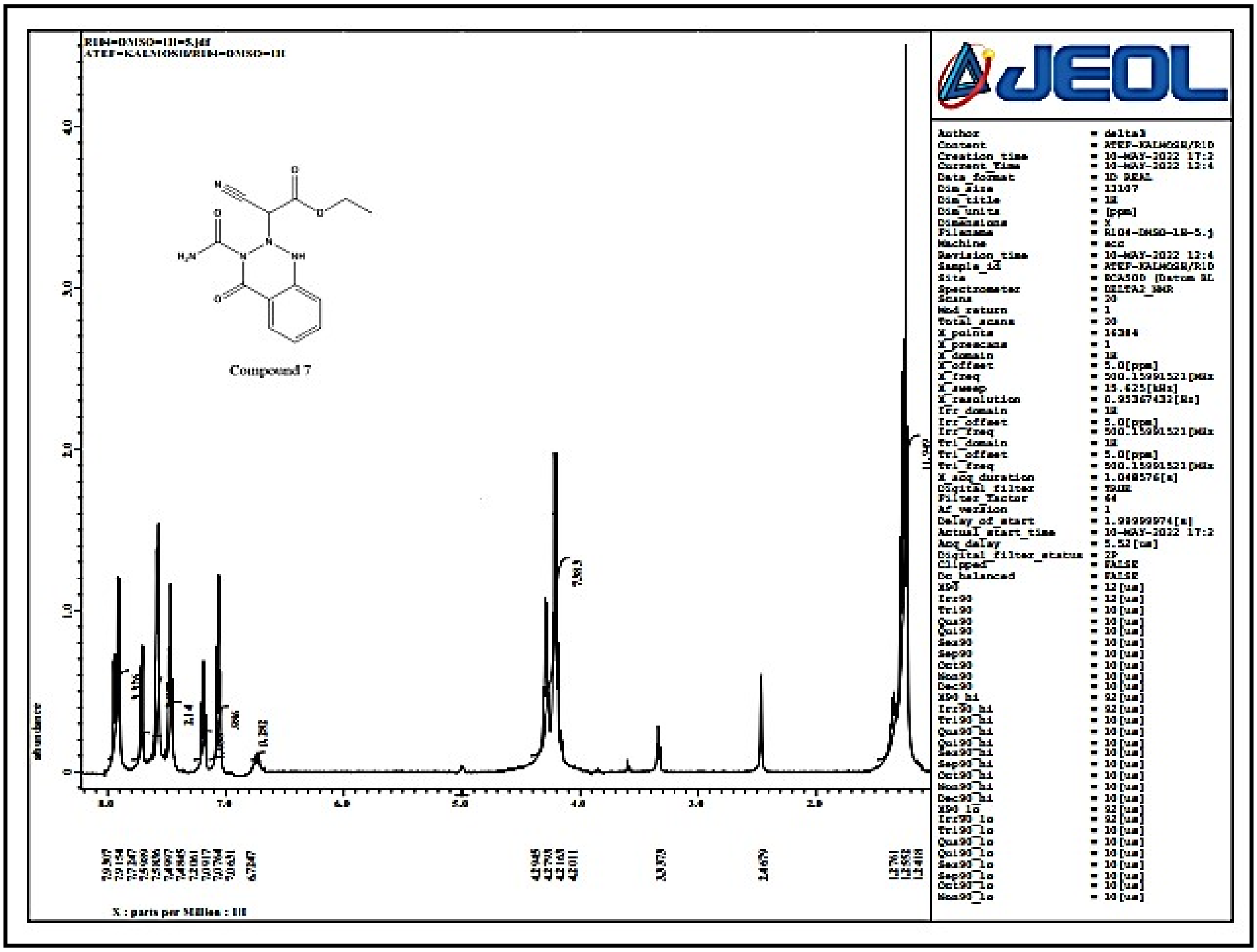

Fig. 12: **^{1}H-NMR Spectrum of compound 7**

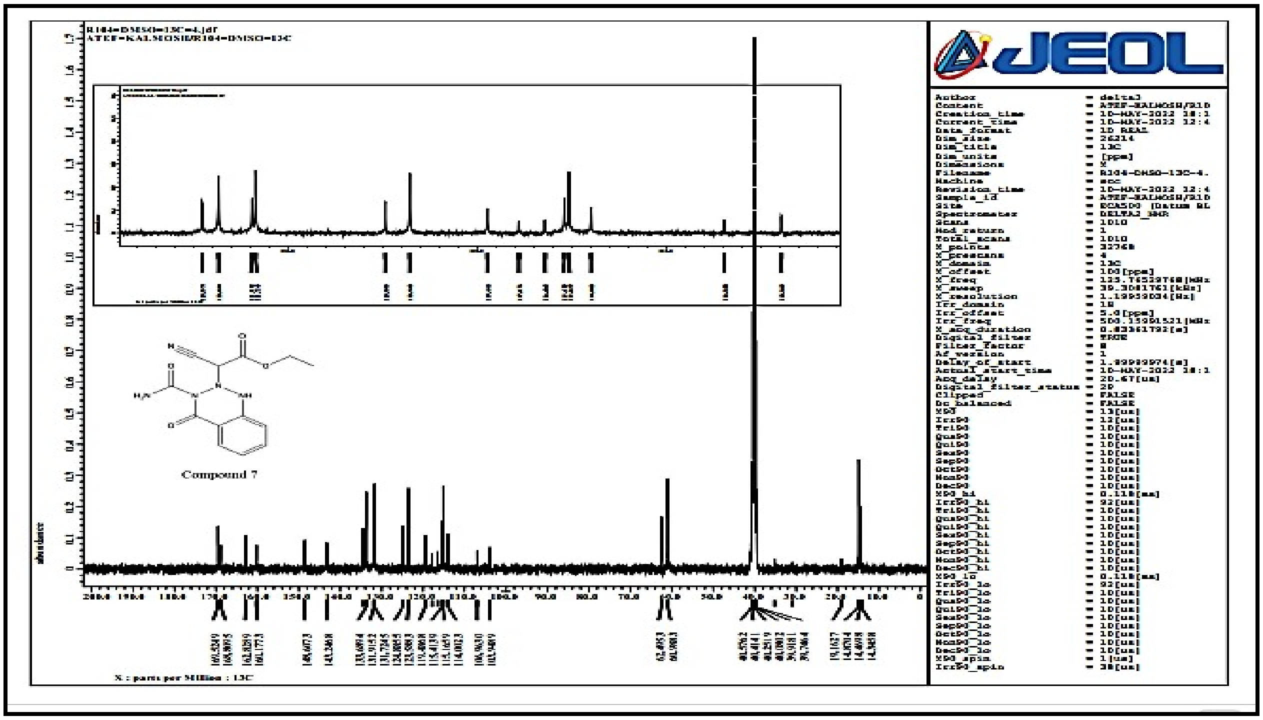

Fig. 13: **^{13}C-NMR Spectrum of compound 7**

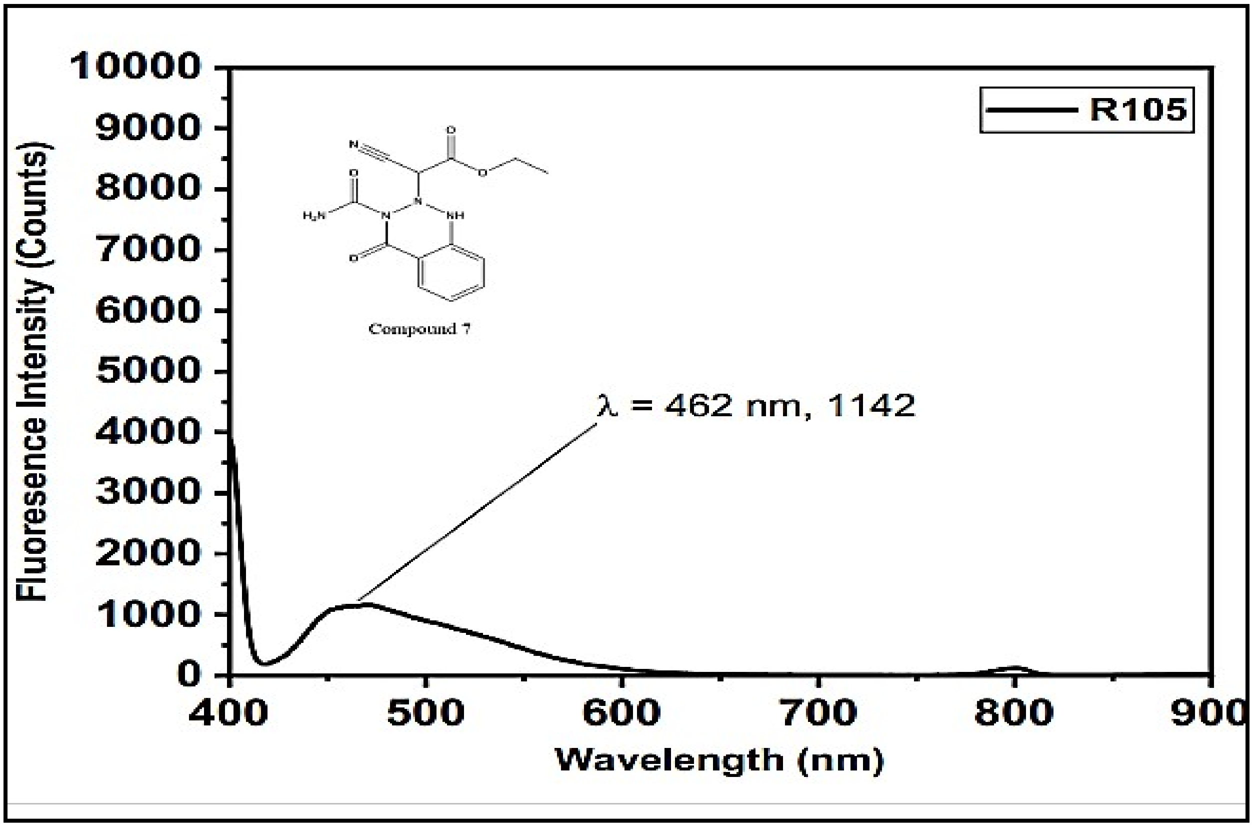

Fig. 14: **Fluorescence spectra of compound 7**

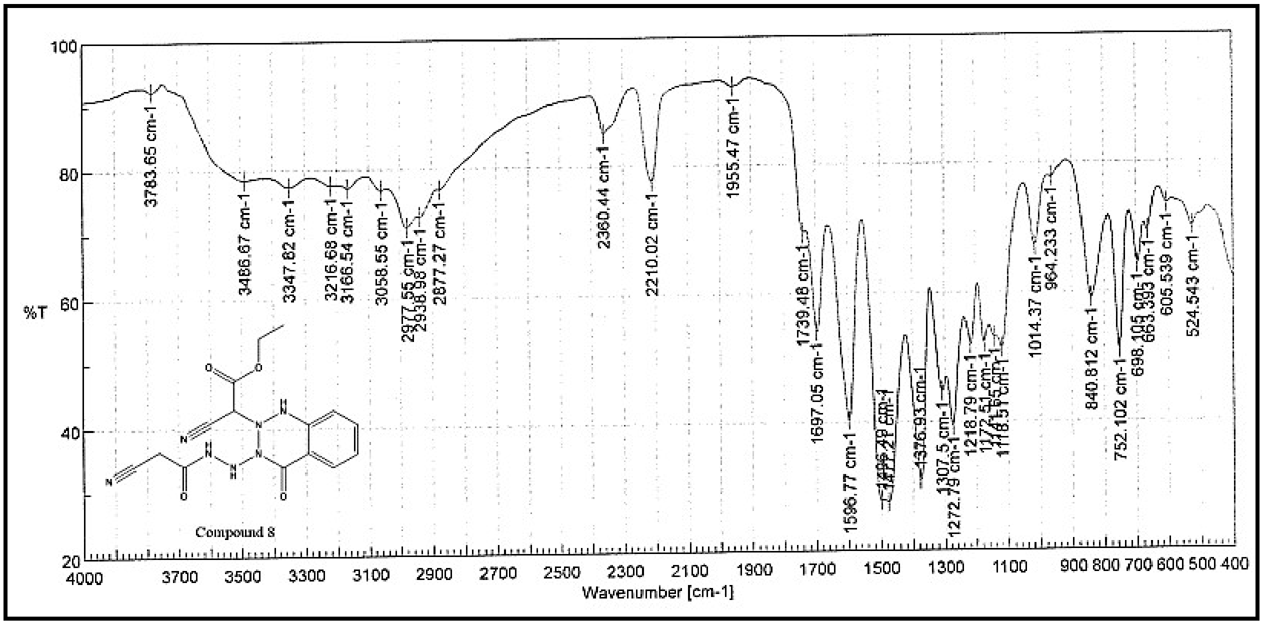

Fig. 15: **IR Spectrum of compound 8**

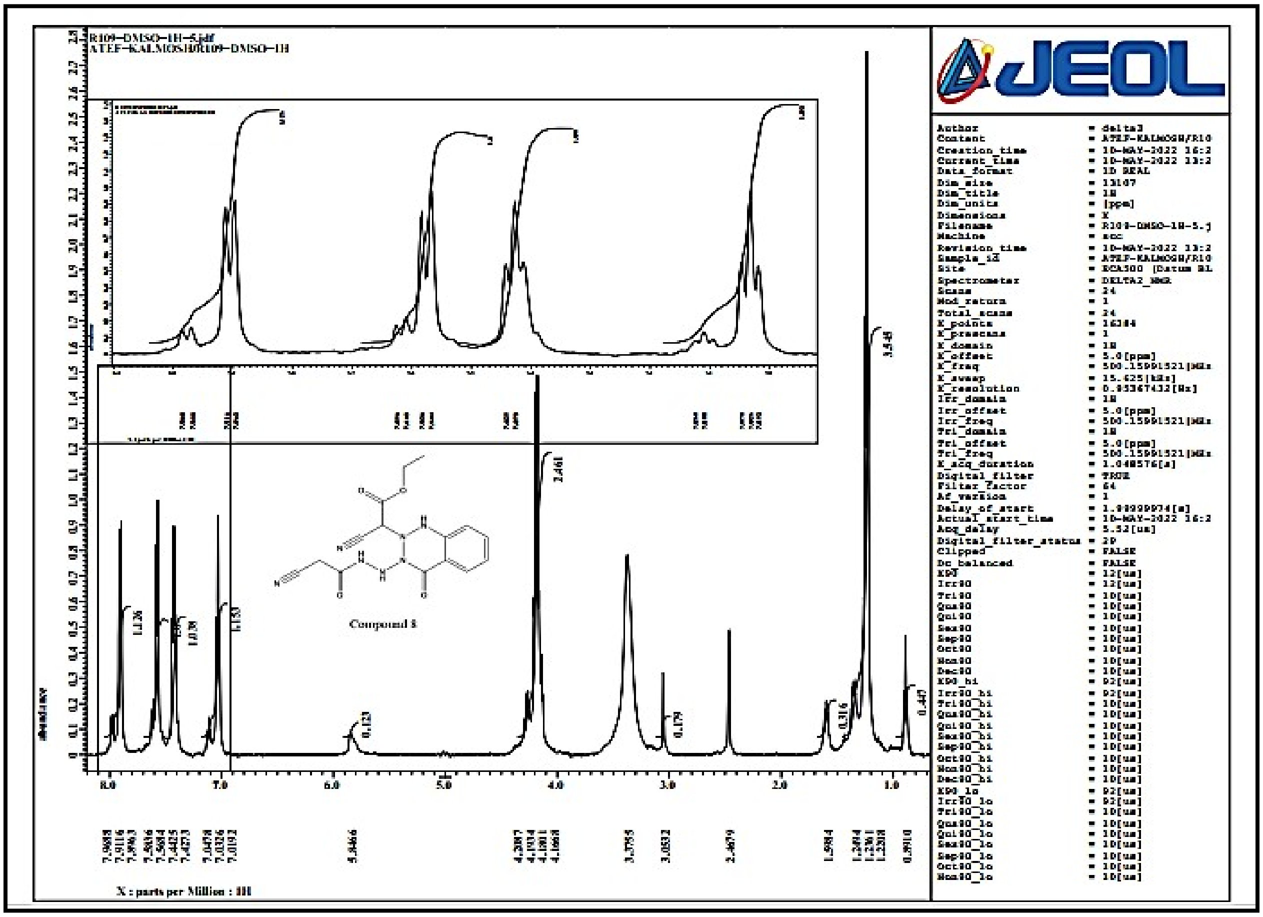

Fig. 16: **^{1}H-NMR Spectrum of compound 8**

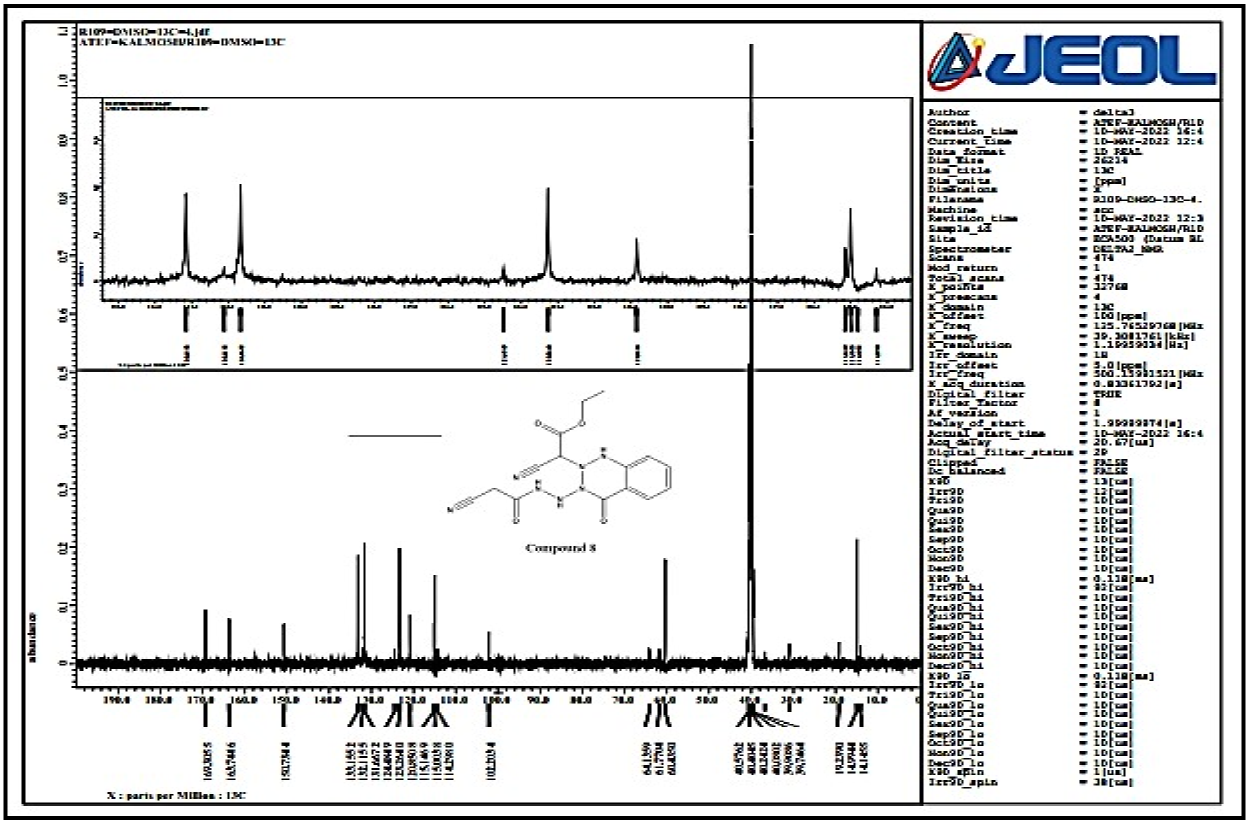

Fig. 17: ^{13}C-NMR Spectrum of compound 8

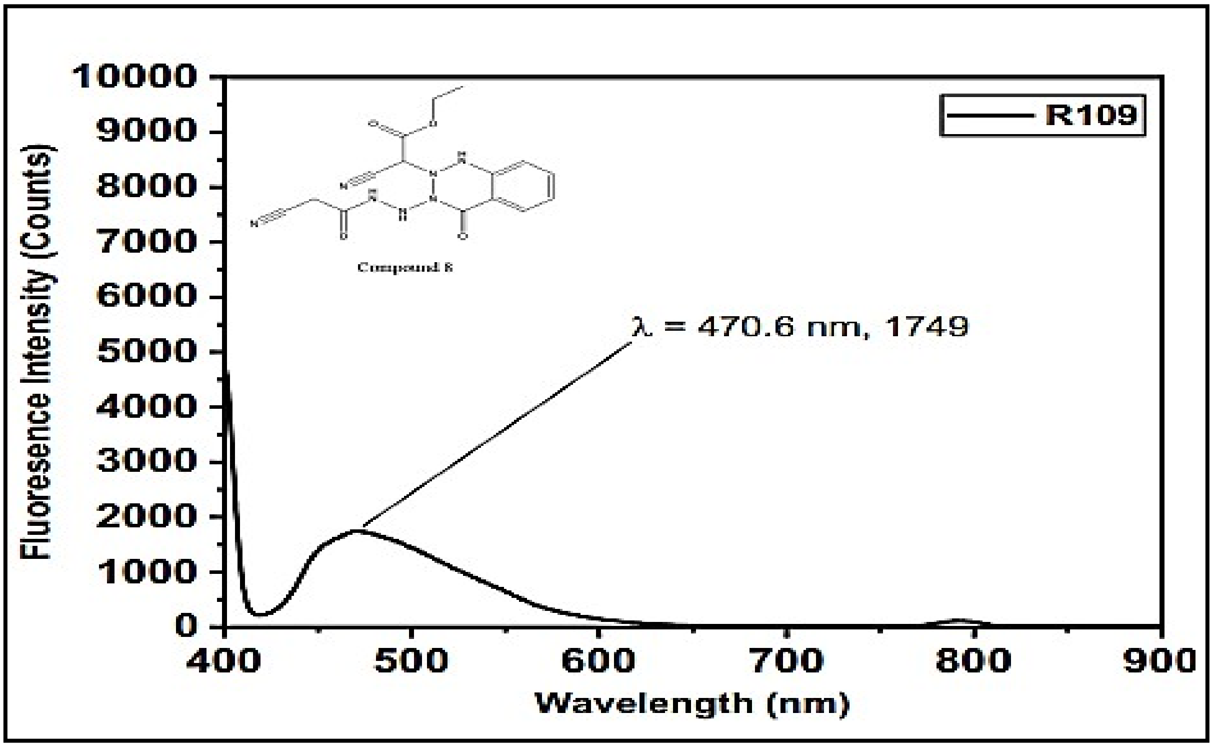

Fig. 18: Fluorescence spectra of compound 8

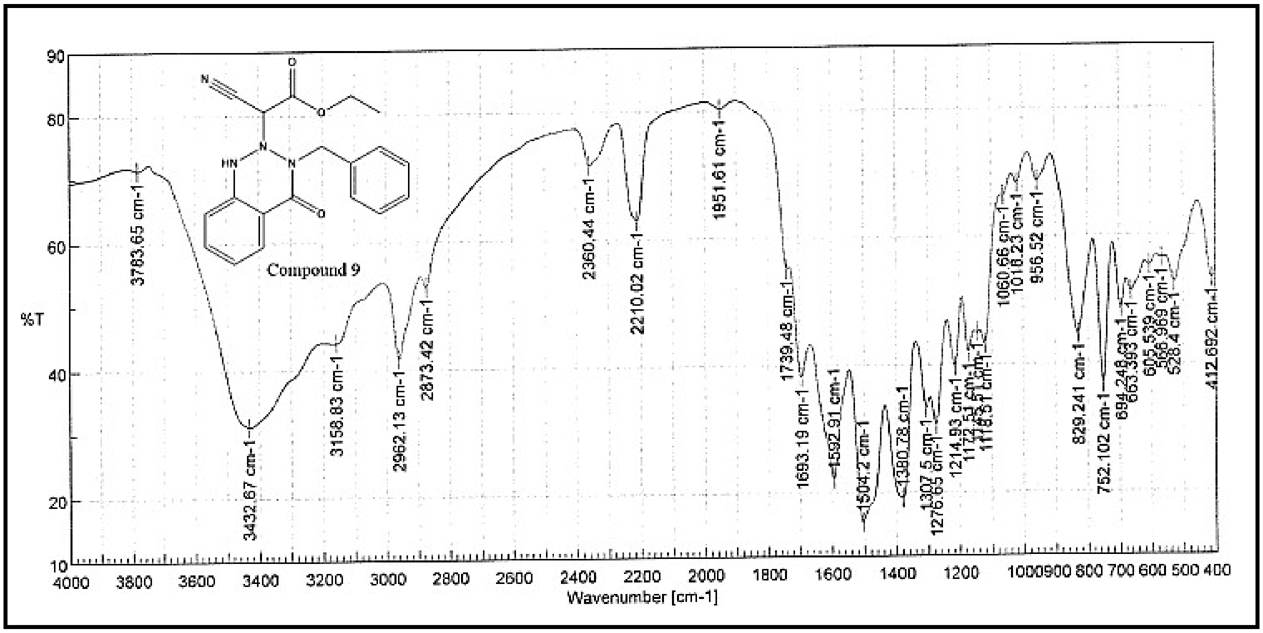

Fig. 19: **IR Spectrum of compound 9**

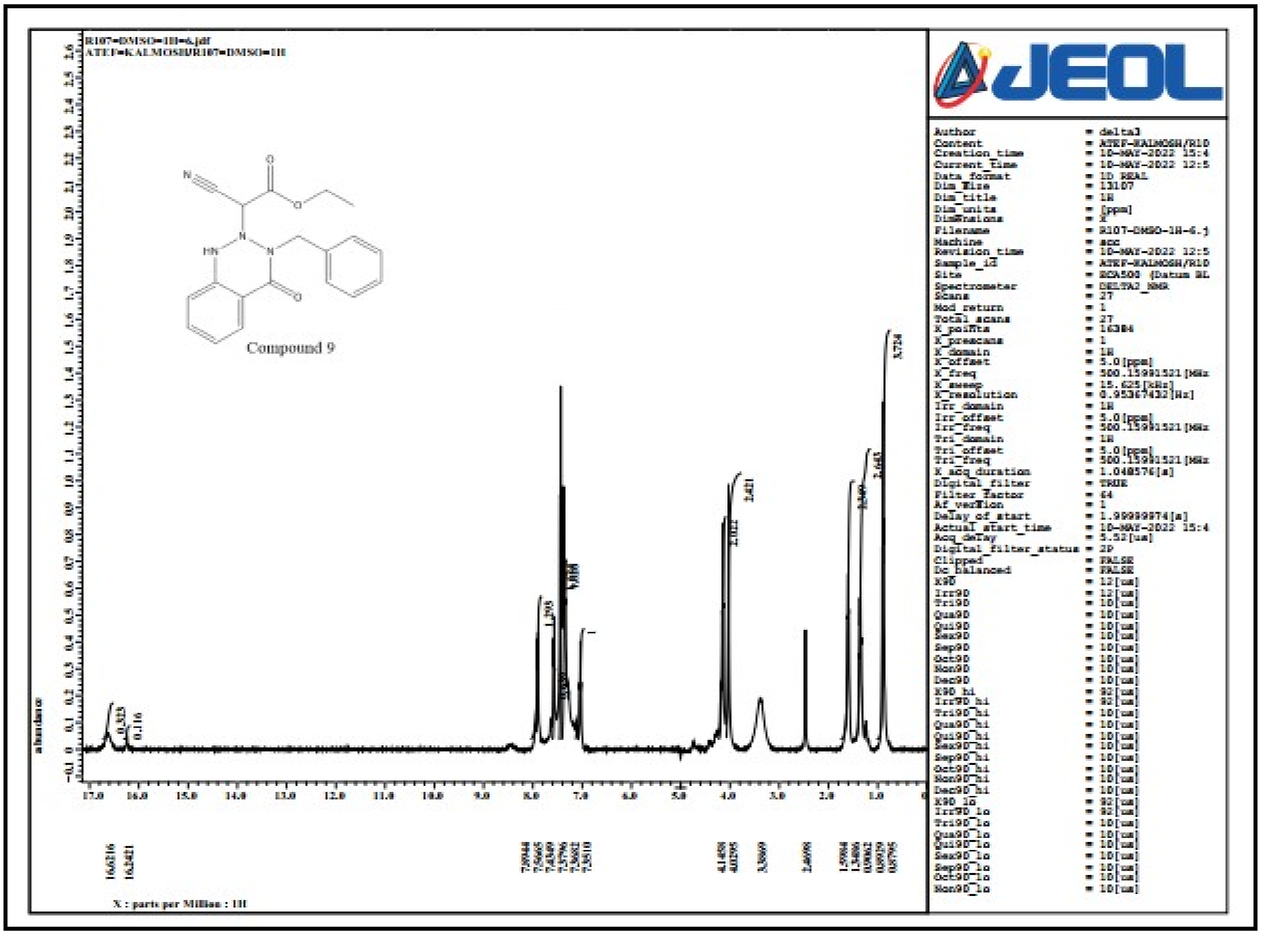

Fig. 20: **^{1}H-NMR Spectrum of compound 9**

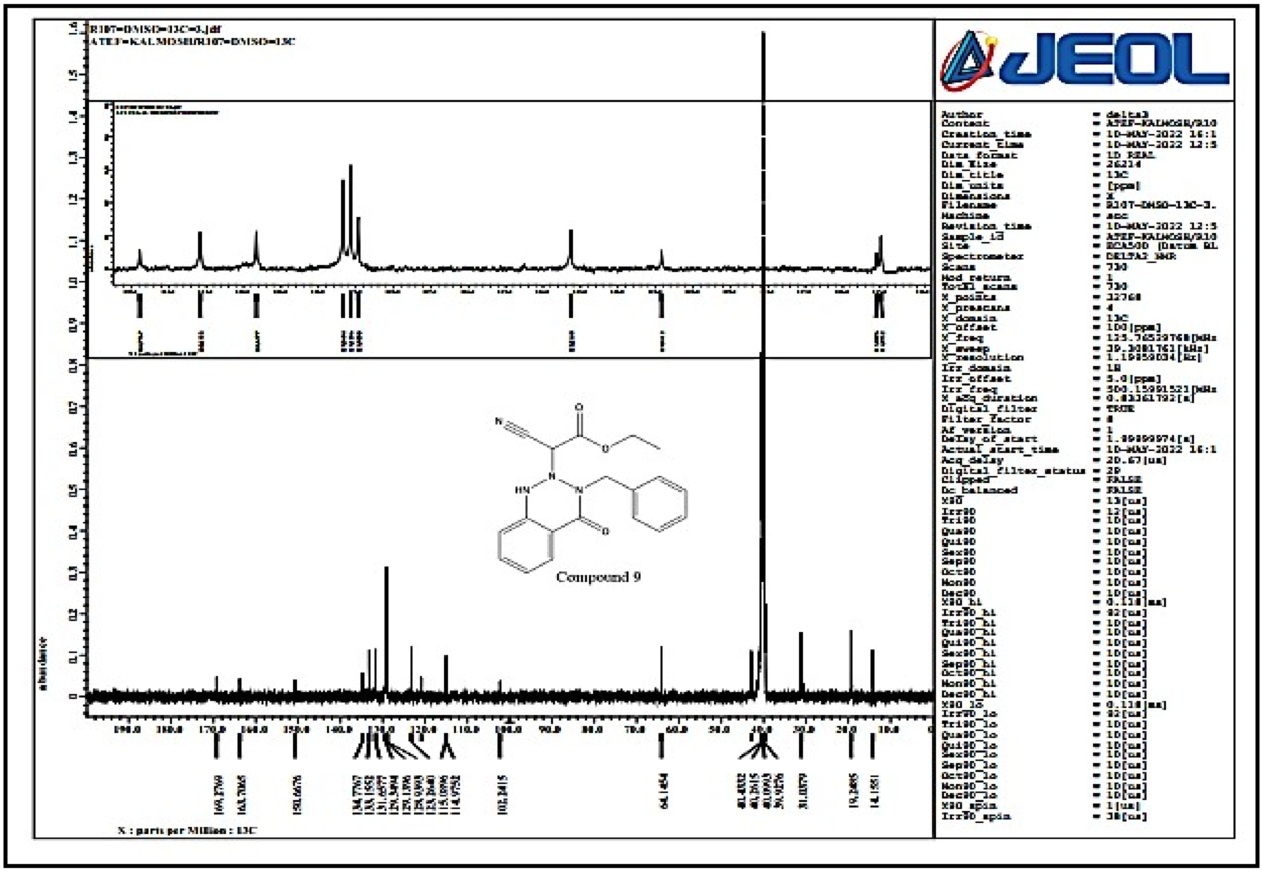

Fig. 21: ^{13}C-NMR Spectrum of compound 9

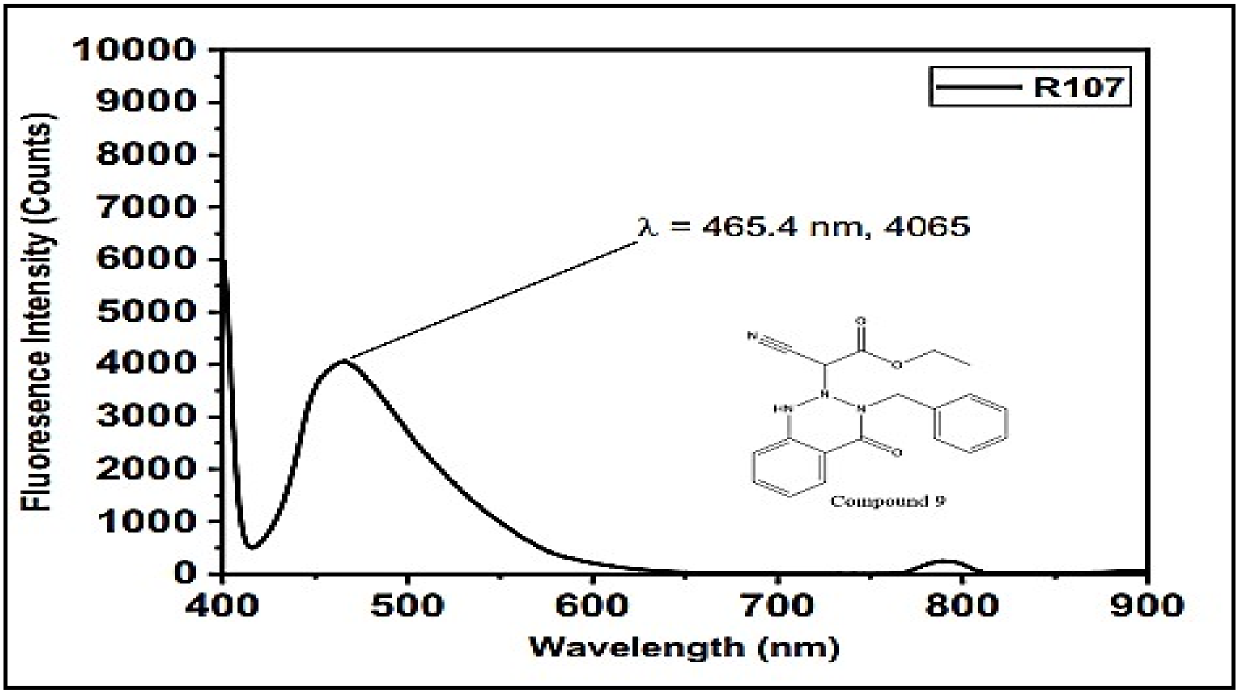

Fig. 22: Fluorescence spectra of compound 9

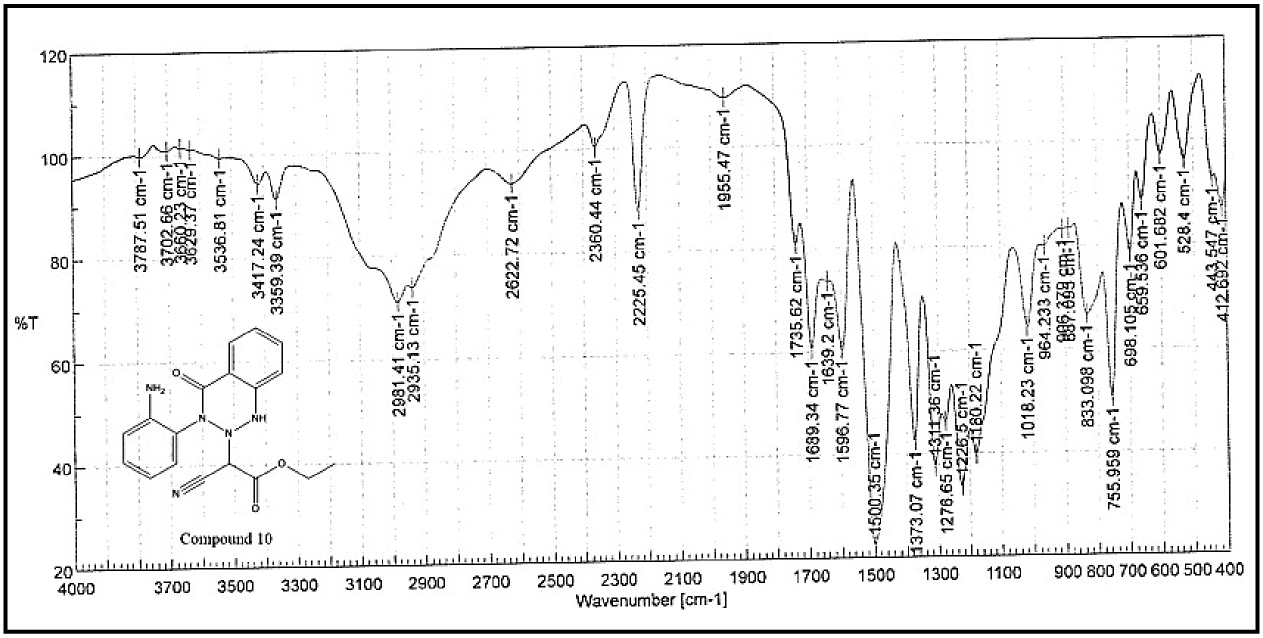

Fig. 23: **IR Spectrum of compound 10**

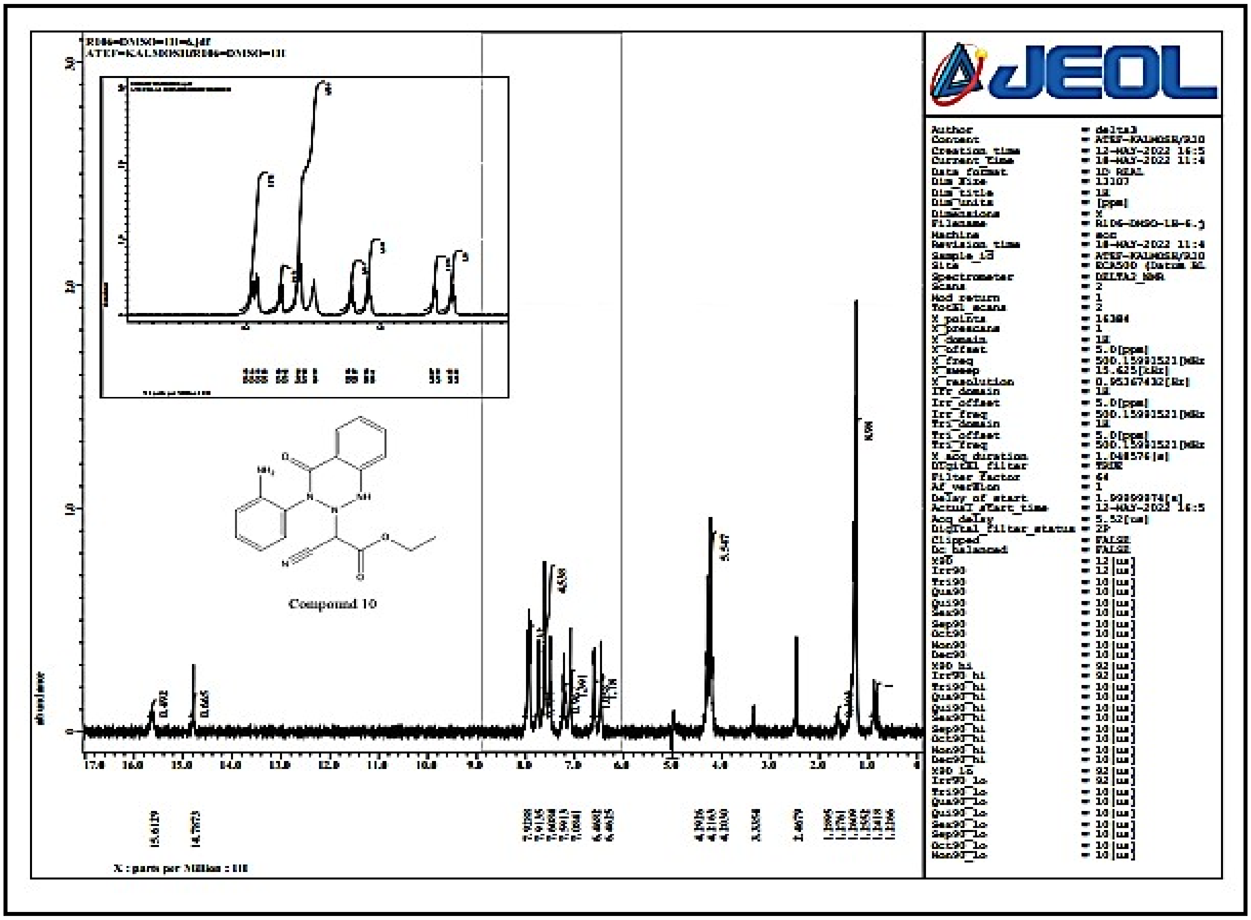

Fig. 24: **^{1}H-NMR Spectrum of compound 10**

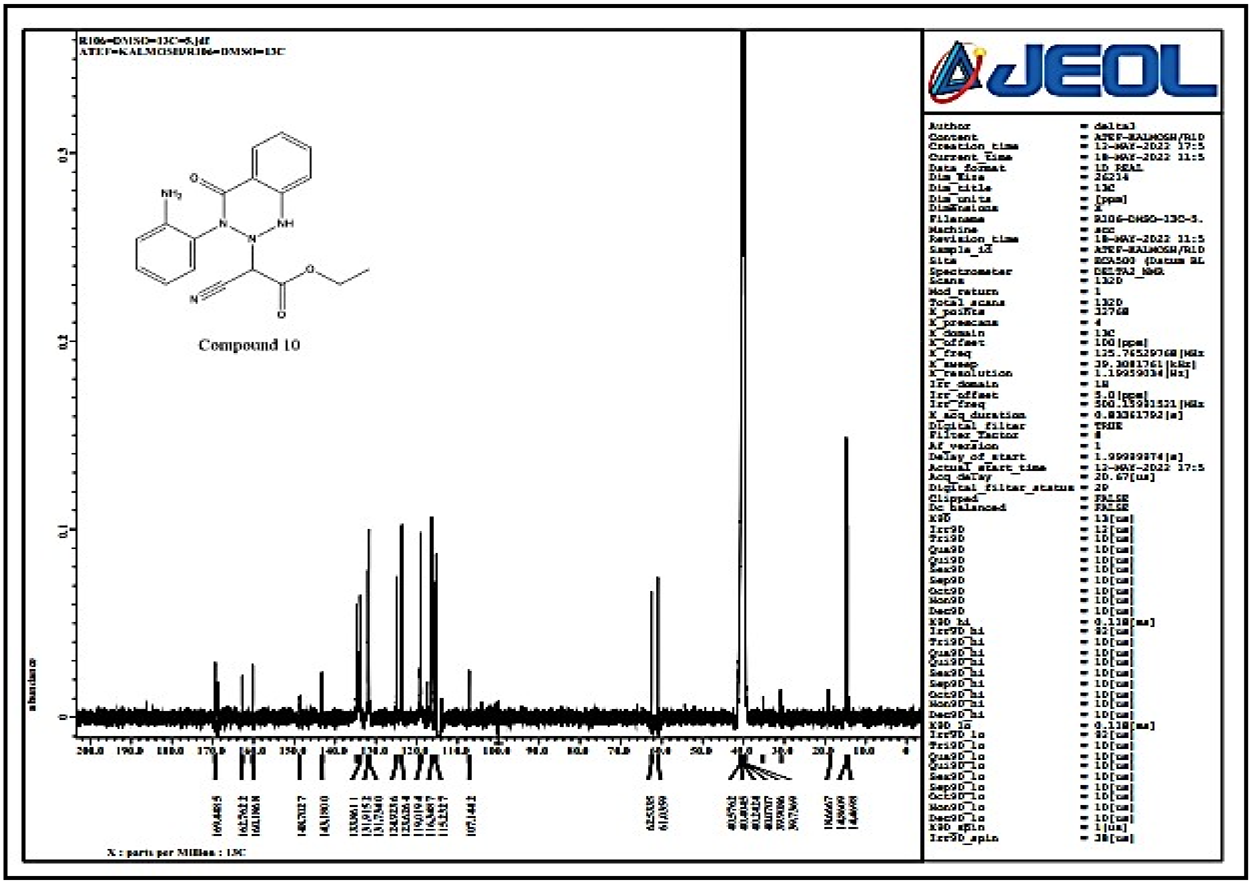

Fig. 25: **^{13}C-NMR Spectrum of compound 10**

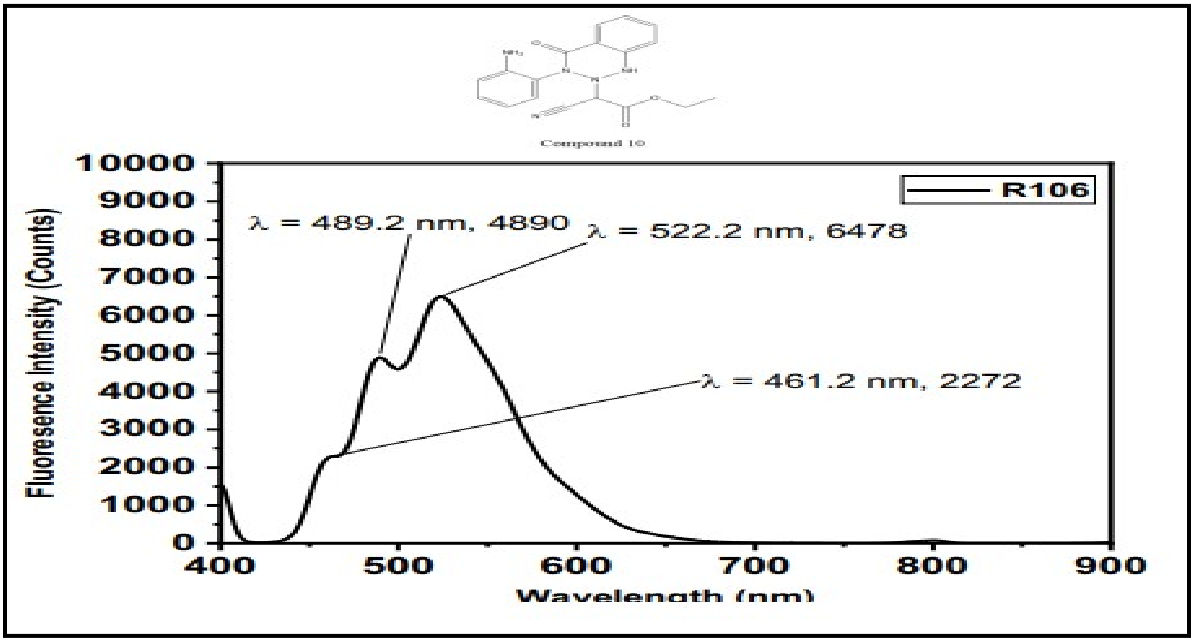

Fig. 26: **Fluorescence spectra of compound 10**

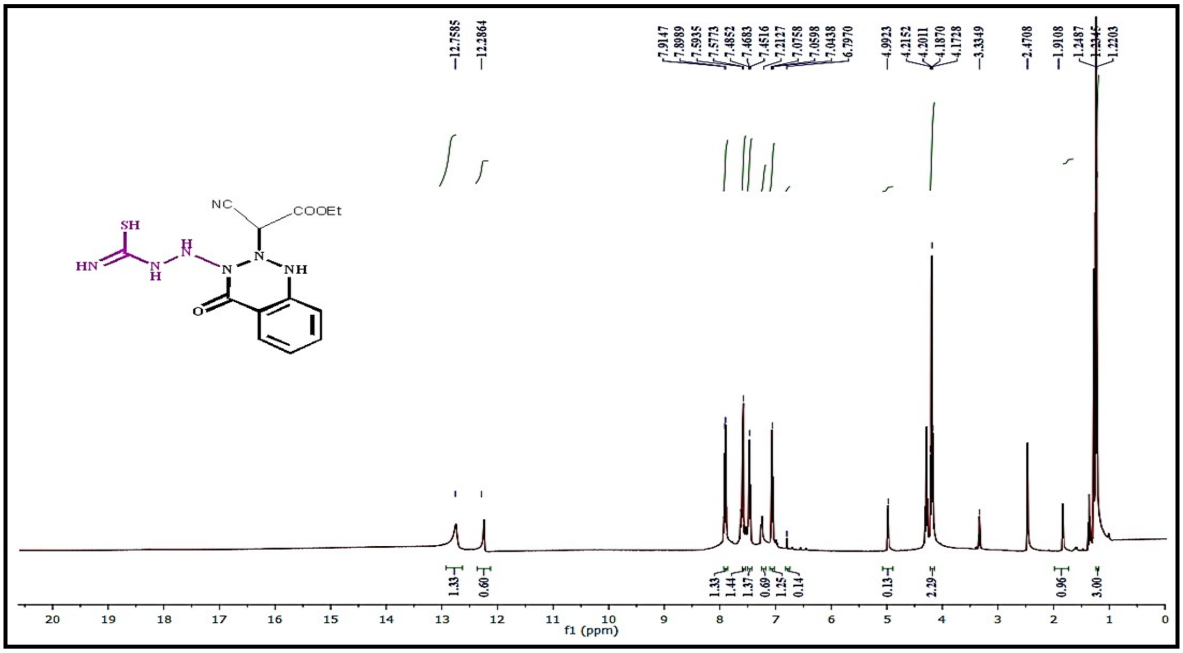

Fig. 27: **^{1}H-NMR Spectrum of compound 12**

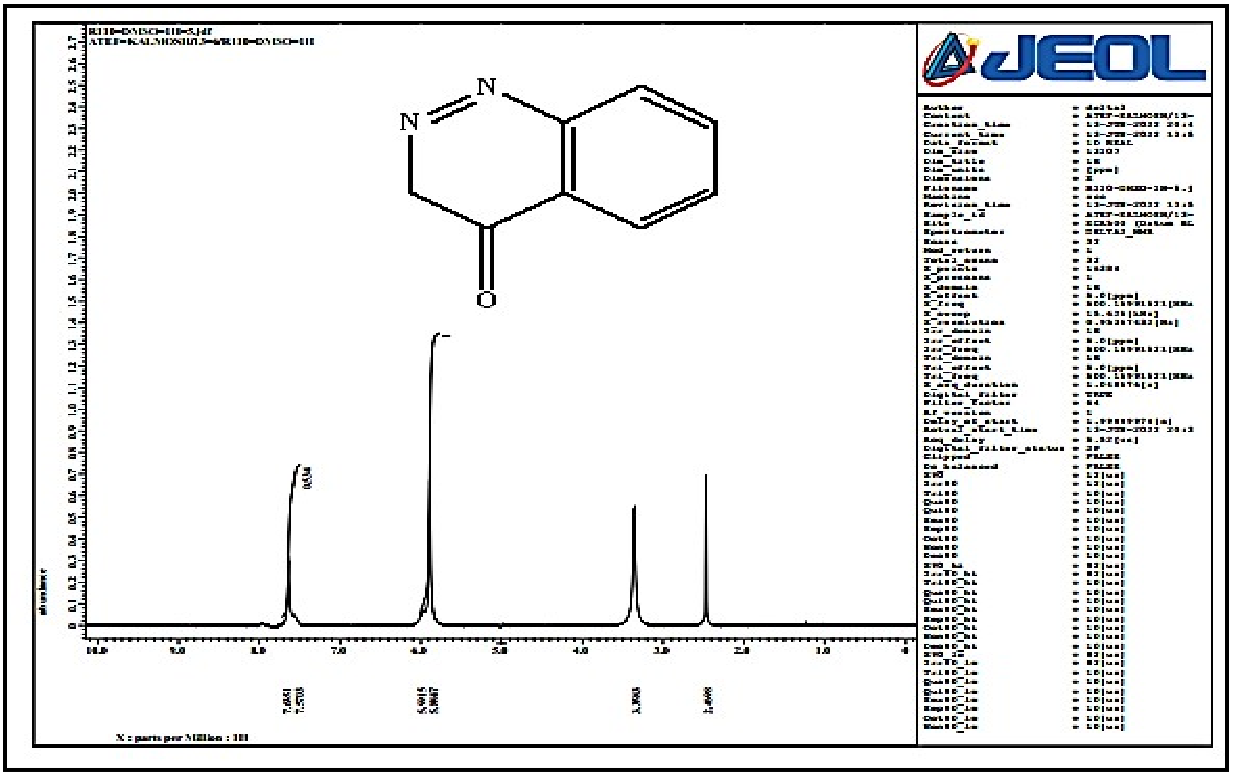

Fig. 28: **^{1}H-NMR Spectrum of compound 14**
